# A Phenotypic Profile of the *Candida albicans* Regulatory Network

**DOI:** 10.1371/journal.pgen.1000783

**Published:** 2009-12-24

**Authors:** Oliver R. Homann, Jeanselle Dea, Suzanne M. Noble, Alexander D. Johnson

**Affiliations:** 1 Department of Microbiology and Immunology, University of California San Francisco, San Francisco, California, United States of America; 2 Department of Medicine, Infectious Diseases Division, University of California San Francisco, San Francisco, California, United States of America; 3 Department of Biochemistry and Biophysics, University of California San Francisco, San Francisco, California, United States of America; The University of North Carolina at Chapel Hill, United States of America

## Abstract

*Candida albicans* is a normal resident of the gastrointestinal tract and also the most prevalent fungal pathogen of humans. It last shared a common ancestor with the model yeast *Saccharomyces cerevisiae* over 300 million years ago. We describe a collection of 143 genetically matched strains of *C. albicans*, each of which has been deleted for a specific transcriptional regulator. This collection represents a large fraction of the non-essential transcription circuitry. A phenotypic profile for each mutant was developed using a screen of 55 growth conditions. The results identify the biological roles of many individual transcriptional regulators; for many, this work represents the first description of their functions. For example, a quarter of the strains showed altered colony formation, a phenotype reflecting transitions among yeast, pseudohyphal, and hyphal cell forms. These transitions, which have been closely linked to pathogenesis, have been extensively studied, yet our work nearly doubles the number of transcriptional regulators known to influence them. As a second example, nearly a quarter of the knockout strains affected sensitivity to commonly used antifungal drugs; although a few transcriptional regulators have previously been implicated in susceptibility to these drugs, our work indicates many additional mechanisms of sensitivity and resistance. Finally, our results inform how transcriptional networks evolve. Comparison with the existing *S. cerevisiae* data (supplemented by additional *S. cerevisiae* experiments reported here) allows the first systematic analysis of phenotypic conservation by orthologous transcriptional regulators over a large evolutionary distance. We find that, despite the many specific wiring changes documented between these species, the general phenotypes of orthologous transcriptional regulator knockouts are largely conserved. These observations support the idea that many wiring changes affect the detailed architecture of the circuit, but not its overall output.

## Author Summary

A key goal in the understanding of the biology of an organism is the description of the regulatory networks that control the expression of its genes. Changes in gene expression result in new cellular phenotypes that can be acted upon by evolutionary forces to influence the configuration of these networks. We have developed a phenotypic description of the transcriptional regulatory networks of the major fungal pathogen of humans, *Candida albicans*, by individually deleting genes that encode transcriptional regulators and observing the resulting phenotypes in a variety of environmental conditions. This approach provides insight into the biological roles of many previously uncharacterized regulators, and allows us to assign groups of regulators to specific biological roles, many of which are relevant to pathogenesis. For example, we identified groups of regulators that influenced sensitivity to antifungal drugs, the ability to acquire iron (a challenge for organisms in a human host), and the ability to form complex multi-cellular colonies. Our results also allow us to analyze how the phenotypes associated with transcriptional regulators change as organisms diverge. A comparison of *C. albicans* data with that from the well-characterized yeast *S. cerevisiae* revealed strong phenotypic conservation between related transcriptional regulators, despite the more than 300 million years which separate the species.

## Introduction

The transcriptional networks that orchestrate gene expression are complex. Even in single-celled organisms, these networks must specify different cell types, must coordinate responses to different external cues, and must maintain homeostasis in a constantly changing environment. The evolution of such networks occurs by numerous mechanisms, including gains, losses, and modifications of transcriptional regulators and the DNA sequences they recognize (cis-regulatory sequences).

With over forty genomes sequenced, the ascomycete fungi are highly amenable to detailed study of regulatory network evolution. The wealth of data for the model organism *Saccharomyces cerevisiae* serves as a particularly strong basis of comparison. In this paper, we broadly explore transcription networks in *Candida albicans*, the most prevalent fungal pathogen of humans, and compare them to those in *S. cerevisiae*. These two organisms last shared a common ancestor some 300–900 million years ago [1],[2], and, in terms of coding sequences, their genomes are approximately as divergent as those of fish and humans [3].

Previous studies have documented similarities in transcriptional wiring between *S. cerevisiae* and *C. albicans*, but have also revealed major differences. In some cases it has been possible to trace plausible evolutionary pathways for these changes [4]–[6]. In these latter studies, a specific transcriptional regulator or set of target genes was typically studied in detail in both organisms. Here we analyze a much larger number of transcriptional regulators (TRs) in *C. albicans*, 143 in all. We monitored a broad spectrum of phenotypes produced when each TR was knocked out by homologous recombination, and we were thus able to interpret the phenotypes of each mutant in the context of the phenotypes observed across the whole mutant collection.

Since *C. albicans* is a diploid organism, two rounds of gene disruption were required to produce each deletion mutant. Because unlinked mutations can occur during the knockout procedure, two or more independent knockout strains were constructed for each TR. Overall, 317 strains were created, representing 143 TRs. Each strain was carefully vetted to ensure that both copies of the appropriate gene had been eliminated. The strain collection was then assayed using 55 different growth conditions to provide an expansive set of phenotypic data. Although a smaller library of TRKOs has been constructed in *C. albicans* [7], extensive phenotyping has not been reported, as the library was designed primarily for preliminary genetic screens.

We believe the extensive strain collection described here, along with the phenotypic data, will be an important resource to the *C. albicans* community. Specific functions can now be assigned to many transcriptional regulators that were previously uncharacterized. Moreover, investigators studying various aspects of *C. albicans* biology, especially those that relate to issues associated with the human host (e.g. drug resistance, morphological variation, iron acquisition), can immediately identify the TRs that control the process of interest and acquire the relevant set of knockout strains. Finally, as demonstrated by Nobile and Mitchell [7], a set of TR deletion strains is a useful reagent for *de novo* genetic screens. Because transcriptional regulators typically control expression of many genes, this approach provides a wide “net” to capture genes involved in any process for which an assay can be devised. Additional strategies (e.g. full-genome chromatin immunoprecipitation) can then be used to link the transcriptional regulator to its target genes. The high level of quality control and the representation of each regulator by at least two independent knockout strains make our deletion set especially well-suited for such genetic screens.

Our large set of phenotypic data, when compared with the comprehensive sets of data generated for *S. cerevisiae* [8]–[14], allows for the first systematic cross-species comparison of the phenotypic conservation of TR function. To ensure the most meaningful comparison between orthologous TRs, we repeated the phenotypic studies on a subset of *S. cerevisiae* TR deletion strains using the same conditions that were applied to the *C. albicans* strains. We provide a systematic analysis of phenotypes associated with orthologous *S. cerevisiae* and *C. albicans* TRs, and, despite the numerous examples of transcriptional rewiring documented to have occurred since the species diverged, we find a high degree of phenotypic conservation.

## Results/Discussion

### Construction and phenotypic profiling of a transcriptional regulator knockout library

The list of candidate genes for inclusion in our transcriptional regulator knockout (TRKO) library was compiled from multiple sources (Dataset S1; [7], [15]–[22]). We defined transcriptional regulators as any protein that binds DNA in and around a gene and influences its transcription rate. We placed an emphasis on proteins with sequence-specific DNA binding domains, and did not include proteins that influence the transcription of most genes in the cell (e.g. histones, subunits of mediator, and the general transcription factors).

**Figure 1:**
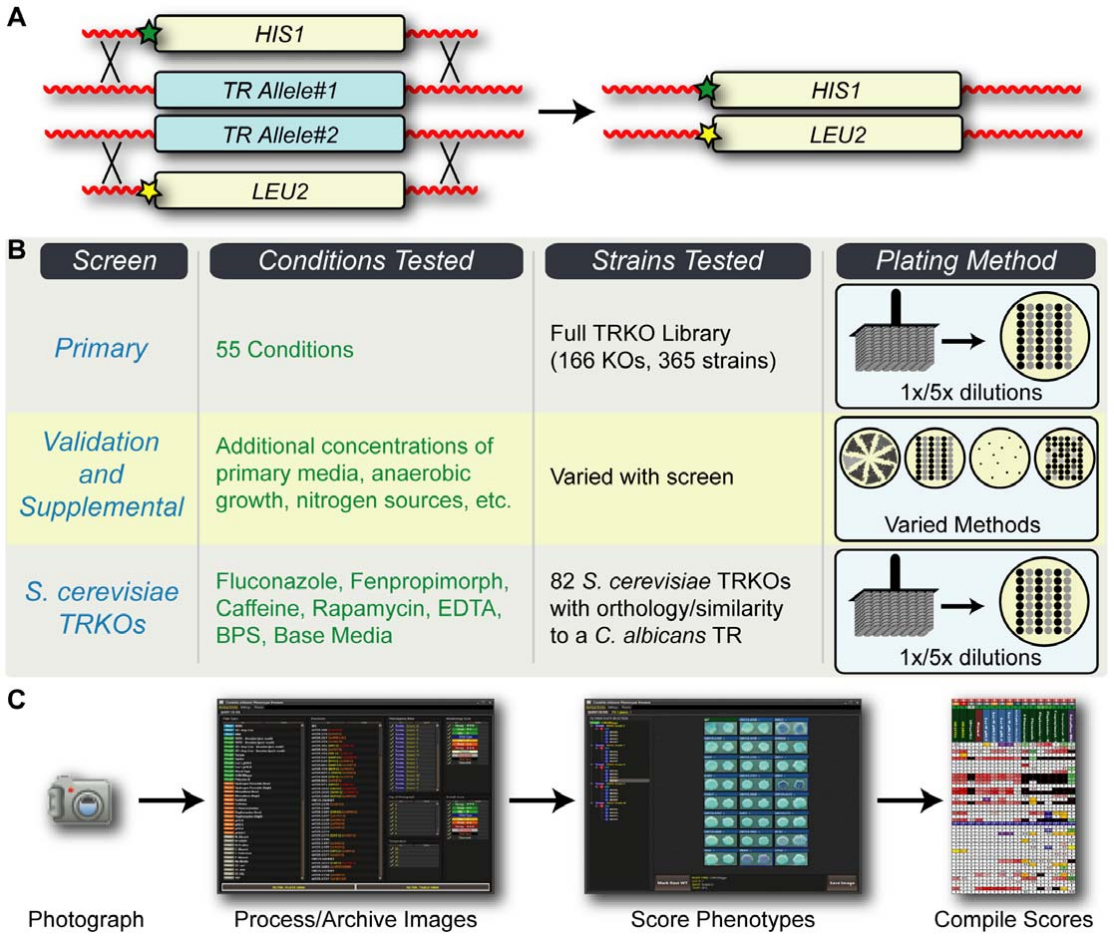
Overview of experimental design. (A) 166 transcriptional regulators were knocked out using homologous recombination. Signature-tagged markers (stars) were included for use in future analyses. (B) The phenotyping screen consisted of a large primary screen and several smaller supplemental screens. (C) Photographs of phenotyping media were processed, archived, and scored using custom Java software. Phenotyping scores were then compiled for each combination of knockout and phenotyping medium and exported to a spreadsheet for further analysis.

To create the TRKO strains, we utilized a fusion-PCR based approach [23] that employs long stretches of flanking homology to maximize recombination (Figure 1A; see Materials and Methods). *C. albicans* is diploid, and the construction of each knockout strain thus required two rounds of gene disruption. Although not used in this study, signature tags were incorporated into each knockout strain to enable strains to be screened in groups. Because it is not straightforward to perform back-crosses with *C. albicans* [24] (and thereby ensure that a given phenotype segregates with a gene disruption), we created two fully independent knockout strains for each TR. This strategy greatly increases the likelihood that a phenotype observed in both strain isolates resulted from the gene knockout rather than an unrelated mutation that arose during the gene disruption procedure. This is an important consideration, as we estimate that as many as 10% of gene knockouts have additional mutations that produce at least moderate phenotypes (see Text S2). Our approach, coupled with the phenotypic screen described below, yielded high-quality TRKOs for 143 of the 184 TRs in our original list. To be classified as high-quality, two independently derived knockout strains must exhibit the same set of phenotypes. (In some cases, additional isolates were created to resolve inter-isolate inconsistencies.) An additional 23 TRKOs are included in our collection, but are classified as lower confidence. In some cases (11 TRKOs) only a single deletion strain was obtained, and in others (12 TRKOs), the independent isolates produced overlapping but distinct phenotypes. The combined collection containing the 143 high-confidence KOs and the 23 lower-confidence KOs contains 166 KOs, represented by 365 total strains. In the following discussions, we focus on the high-confidence TRKOs.

Phenotypic profiles for each TRKO were established by a large primary screen of 55 conditions augmented by a series of case-by-case supplemental screens (Figure 1B; Materials and Methods). Phenotyping media were selected to probe a broad spectrum of regulatory networks. We used nutritional cues, temperature, signals that induce morphological changes, antifungal drugs, and a variety of stress conditions. When possible, drug/toxin/nutrient concentrations were calibrated such that both impairment and enhancement of growth relative to wild-type could be observed. A summary of the media utilized in this study, including commentary on their known properties (e.g. modes of action of drugs), is provided as supporting information (Text S1).

**Figure 2:**
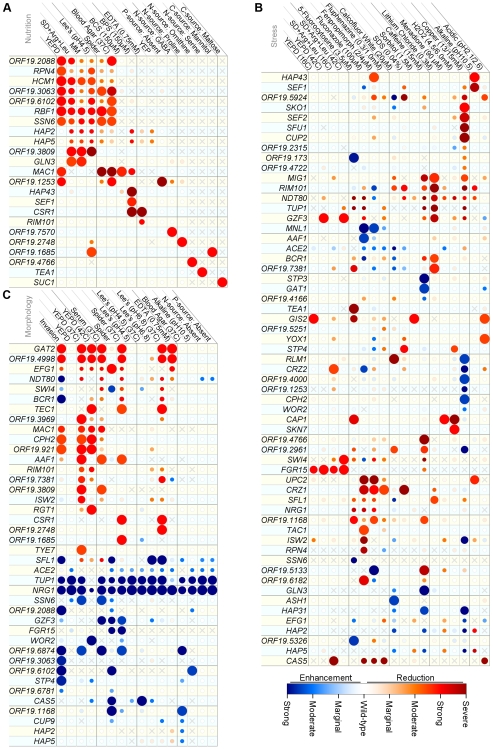
Phenotypes of *C. albicans* transcriptional regulator knockout strains. (A) Nutrient and base media growth phenotypes. (B) Stress media growth phenotypes. (C) Colony morphology phenotypes. The data shown are predominantly from the primary screen, and in some cases scores from two or more similar media have been condensed to a single column (as described in Text S2). Phenotype scores are represented by the color of the indicated circle, and the intensity of the color represents the strength of the phenotype. White circles represent a phenotype that is indistinguishable from wild-type. Blue and red circles represent either an enhancement or reduction, respectively, of growth (A and B) or colony morphology (wrinkling or invasion; (C) relative to wild-type. Dark red circles (‘severe’ phenotype; growth phenotypes only) indicate that the TRKO strain failed to show any growth, while under the same conditions the wild-type strain exhibited significant growth. A grey ‘X’ represents missing data due to poor growth on the underlying base medium, phenotypic inconsistencies, or absence of testing for the given combination of strain and medium. The size of the circles reflects the strength of the specificity score for the given phenotype. High specificity scores (large diameter) suggest that the TR is a primary regulator of the circuits probed by the medium and the TR (see text and Text S2).

In the primary screen, independent isolates of each TRKO were plated as 1× and 5× dilutions on a wide range of solid media using a bolt-replicator and then photographed several times over the course of growth. These images were processed and archived using custom Java software (Figure 1C) and scored for growth and morphological phenotypes by comparison to a wild-type control strain included on the same plate. This approach generated over 100,000 individual growth and morphology scores, which were then merged – across time-points and across the knockout isolates of each TR – into single growth and morphology scores for each TRKO on each growth medium (Dataset S2). The scoring system classified the strength of the reduction or enhancement of both growth and morphology relative to wild-type (see Text S2 and Figure 2A legend). Because we observed growth of all strains at two different dilutions and repeatedly over several days, we could easily score subtle phenotypes that might not have been apparent from a single concentration and time-point.

We paid particular attention to colony morphology as a phenotype. On most solid media, colonies of *C. albicans* are composed of three types of cells: budding yeast (round cells), pseudohyphae (strings of ellipsoidal cells that remain attached to one another following cell division), and hyphae (highly elongated cylindrical cells that remain attached following cell division). All three forms are also found in infected tissues, and the transition between these forms is key for normal pathogenesis (reviewed by Biswas et al. [25] and Whiteway and Bachewich [26]). Colony morphology serves as a sensitive assay for differences in the way cells regulate the transition between the three morphological forms. By observing the collection of TRKO mutants on a variety of media over time courses of several days, we were able to identify a broad spectrum of differences in colony morphology.

All images generated in this study will be made available via a Java application hosted by the Candida Genome Database (CGD) [16]. We have also hand-annotated a phenotypic overview of each TRKO (Dataset S2).

### Identifying key associations between phenotype and regulator in a large dataset

The primary phenotypic screen identified at least one moderate phenotype for over 50% of the tested TRKOs and at least one strong phenotype for over 40% of the tested TRKOs. Many of these transcriptional regulators were completely uncharacterized, and this study presents the first direct experimental data relevant to their function.

The phenotypic profiles generated by the primary screen are provided in Figure 2. The assay conditions have been separated into the broad categories of nutrition (Figure 2A), stress (Figure 2B), and morphology (Figure 2C), with the understanding that these categories do not have precisely defined boundaries. The color scale represents a range of phenotype strength from strong enhancement of growth or morphology (blue circles) to strong reduction of growth or morphology (red circles). In order to highlight phenotypes that are more likely to reflect a direct role of a given TR, we scaled the diameter of the circles to reflect a “specificity score”. A high specificity score (large diameter) indicates that the TR deletion shows a strong phenotype under the indicated condition and an overall low level of pleiotropy (i.e. few phenotypes overall) relative to the other TRKOs that exhibited a phenotype under the given growth condition. This approach thus deemphasizes a highly pleiotropic TRKO (small diameter) if other less pleiotropic TRKOs share the phenotype in question. The calculation of the specificity score (Text S2) was conducted independently for enhancement and reduction phenotypes, and only strong phenotypes were considered.

A high specificity score (large diameter circle) serves as a visual marker for those TRs that are likely to control relatively small and discrete circuits. In other words, the TR is likely to regulate a small set of genes whose misregulation in the deletion mutant causes a restricted set of phenotypes. In contrast, a low specificity score could indicate that (1) the TR directly controls many genes involved in many different biological processes, (2) the regulator regulates one or more TRs with higher specificity scores, or (3) that the regulator directly controls a relatively small circuit but that disruption of the circuit causes many indirect phenotypes.

The TRKOs with high specificity scores and strong phenotypes form the basis of much of our analysis. In the following three sections, we discuss, through specific examples, general ways in which the phenotypic profiles can be applied to problems in *C. albicans* biology. Although we cite specific examples as support, we do so primarily to illustrate the generality of these approaches. These three sections are followed by a more focused discussion of the regulation of *C. albicans* morphology. We conclude with a discussion of the evolution of transcriptional circuits based on a comparison of biological roles of orthologous regulators in *C. albicans* and *S. cerevisiae*.

### “Transposing” a well-studied transcription network from *S. cerevisiae* to *C. albicans*

*S. cerevisiae* is a particularly well-studied eukaryotic organism, and observations made in this species have often been used as the starting point for studies in *C. albicans*. This approach has had mixed success; the failures often result from homologous proteins playing markedly different biological roles in the two species. Our results can help reveal the extent to which a transcriptional circuit worked out in detail in *S. cerevisiae* is directly applicable to the understudied species *C. albicans*. As an example, we discuss the collection of *C. albicans* mutants that affected the TOR pathway, a critical regulator of cell growth (reviewed in [27]).

When nutrients are abundant, the TOR pathway promotes cell growth and represses genes involved in the utilization of non-preferred nutrient sources. Conversely, when nutrients are limiting, the TOR pathway slows cell growth and redirects cellular resources to scavenge for nutrients. Although components of the TOR pathway are conserved across the eukaryotic lineage, the extent of TOR pathway conservation between *C. albicans* and *S. cerevisiae* was not known with certainty; nor was it known how additional features of *C. albicans* might be connected to the TOR signaling pathway.

The drugs rapamycin and caffeine both inhibit function of the Tor1 kinase [28]–[31], resulting in an artificial signal of cell starvation. In our primary screen, we assayed the deletion collection for sensitivity and resistance to caffeine to identify TRs connected to TOR function. We subsequently tested caffeine-sensitive and -resistant mutants with rapamycin and found a near-perfect correspondence (Dataset S2, and see below), providing additional support for a growing consensus that the primary mechanism of action of caffeine is interference with TOR function rather than disruption of cAMP signaling [29]. The screens identified 22 TRKOs with moderate or strong caffeine (Figure 2B) and rapamycin (Dataset S2) phenotypes.

A detailed analysis of these genes is provided in the supporting materials (Text S3), and here we emphasize four points that emerged from the *C. albicans*-*S. cerevisiae* comparison. First, the core regulatory network governing TOR function is highly conserved between the two species. Specifically, orthologs of five of the six TRs known to interact with Tor1 in *S. cerevisiae* [32] have strong caffeine and rapamycin phenotypes in *C. albicans* (the sixth has no clear ortholog in *C. albicans*; see Text S3 for details). Second, the caffeine screen identified eight additional regulators in *C. albicans* that are homologous to regulators of nutritional pathways in *S. cerevisiae*. These results support the prevailing model that Tor1 signaling is governed by a core regulatory network with additional regulators governing specific nutritional inputs and outputs; these additional regulators appear to be largely the same in *C. albicans* and *S. cerevisiae*. Third, five of the *C. albicans* TRKOs with altered caffeine sensitivity also showed profound alterations in colony morphology, indicating an intimate connection between the TOR pathway and the large cell morphology network (see below). It has been previously reported that rapamycin can both inhibit hyphal formation on solid medium [33],[34] and promote flocculation and aggregation in liquid medium [33] in *C. albicans* [34]. Our results support this connection and further identify the transcriptional regulators likely to mediate it. Finally, although an excellent correspondence between caffeine and rapamycin phenotypes was observed, we did identify one mutant (ΔΔ*orf19.4166*) where this correspondence was lost: the mutant exhibits heightened sensitivity to caffeine, but not rapamycin (Dataset S2). It is possible that this TR regulates genes influencing the import, export, or degradation of caffeine but not rapamycin. Alternatively, this TR may regulate a caffeine-specific cellular target.

In summary, comparing the *S. cerevisiae* TOR regulatory network with homologous regulators in *C. albicans* reveals a strong conserved core pathway that is closely connected to transcriptional circuits governing colony morphology. In general, this approach provides a rapid means of identifying core regulators in *C. albicans*, and in this case it indicates that most of the work on the TOR pathway in *S. cerevisiae* can be directly superimposed onto *C. albicans*.

### Uncovering examples of circuit rewiring

Although the TOR pathway regulators exhibit a high degree of functional conservation between *S. cerevisiae* and *C. albicans*, there are multiple examples of *C. albicans* homologs (and even orthologs) of *S. cerevisiae* transcriptional regulators that have different biological roles in the two species [35],[36]. These case studies illustrate the danger in assigning biological roles to *C. albicans* TRs based solely on homology arguments.

A comparison of the phenotypic data presented here with the extensive sets of data available for *S. cerevisiae* can experimentally validate homology assignments (as for the TOR pathway discussed above); it can also reveal examples of rewiring of a regulatory circuit. As an example of the latter, we consider the regulatory networks governing iron acquisition. The sources and abundance of available iron vary greatly with microenvironment, and iron-acquisition and homeostasis is a special challenge for microorganisms such as *C. albicans* that compete for iron in a mammalian host (reviewed by Sutak et al. [37]).

**Figure 3:**
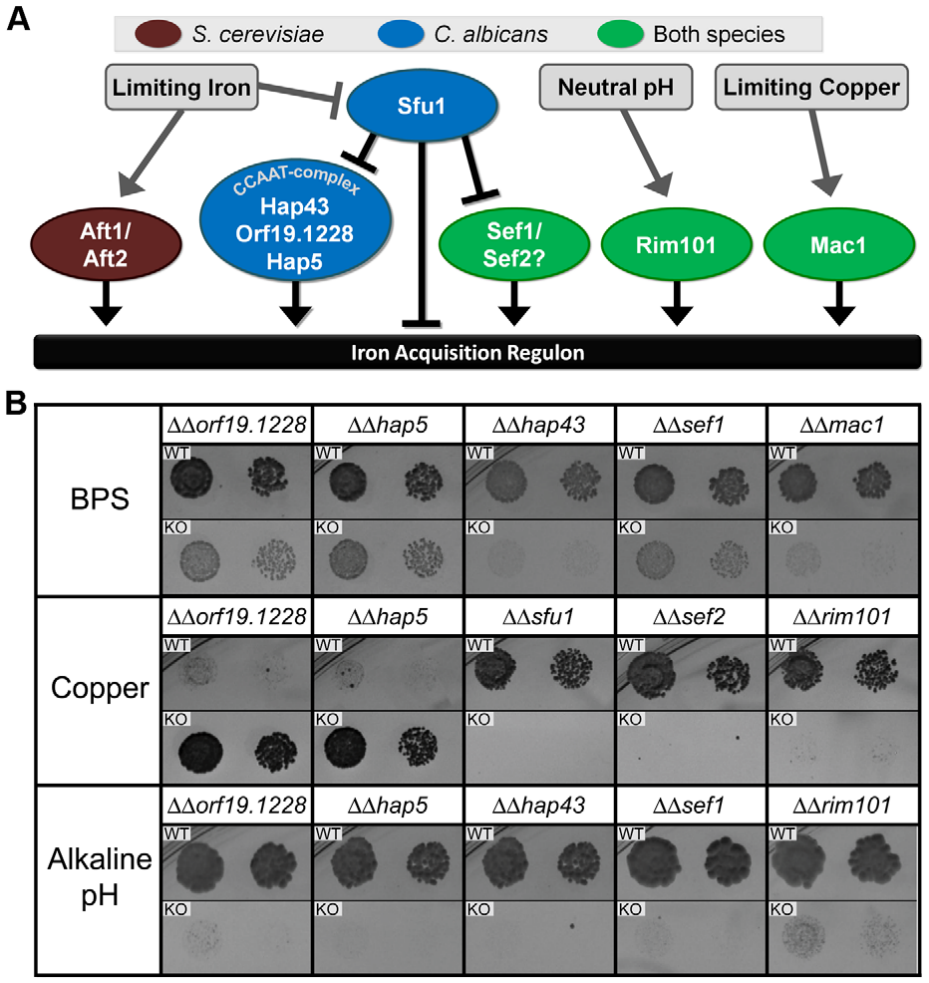
A model of the differences in iron homeostasis regulation between *S. cerevisiae* and *C. albicans*. (A) Each ellipse is colored to reflect whether the indicated gene(s) are believed to play a role in iron homeostasis in *C. albicans* (blue), *S. cerevisiae* (burgundy), or both species (green). (B) *C. albicans* phenotyping data that informed the model are displayed in the bottom panel. These phenotypes include sensitivity to the iron chelator BPS, copper-sensitivity/resistance, and sensitivity to alkaline pH. For each growth condition, only mutants with strong phenotypes are shown (*ΔΔmac1* was not scored on alkaline medium due to poor growth on the YEPD base). Each square phenotype panel displays the wild-type phenotype (1× and 5× dilutions) above the mutant phenotype (the image coloration is inverted to better highlight growth). The panels display the timepoint and medium concentration that showed the most dramatic difference between wild-type and mutant. BPS phenotypes were also assayed for *S. cerevisiae* mutants, and were identical to those of the *C. albicans* orthologs, with two exceptions: (1) the CCAAT-complex mutants were BPS-sensitive in *C. albicans*, but not *S. cerevisiae*, and (2) *Δaft1* and *Δaft2* were BPS-sensitive in *S. cerevisiae* but the likely *C. albicans* ortholog (*ΔΔorf19.2272*) was not BPS-sensitive. Although the *C. albicans ΔΔcsr1* mutant is BPS-sensitive, we have omitted Csr1 because the phenotype is only evident at high BPS concentrations and is likely to reflect a role in zinc homeostasis (see BPS entry in Text S1).

In Figure 3A, we have integrated the data from our phenotypic screen with data from previous studies of iron acquisition in both *S. cerevisiae* (reviewed by [38],[39] and also [9]) and *C. albicans* [40]–[42] to highlight differences in the regulation of iron acquisition and homeostasis between these two species. The data from the screen is based on three growth phenotypes associated with perturbation of iron homeostasis (Figure 3B). The first and most direct phenotype, sensitivity to the iron chelator bathophenanthroline disulfonate (BPS), likely reflects a defect in the iron acquisition circuitry. The second phenotype, sensitivity to elevated copper levels, is linked to iron homeostasis by virtue of the strong inter-connection between copper and iron homeostasis networks: copper is a critical cofactor for high affinity iron uptake [39]. The final phenotype is sensitivity to alkaline pH. Studies in *S. cerevisiae* have established that copper and iron become limiting nutrients in an alkaline growth environment [43].

The phenotypic analysis provides strong support for the idea that the iron acquisition circuit has undergone a major change in regulation since *S. cerevisiae* and *C. albicans* last shared a common ancestor. As shown in Figure 3A, the circuit is positively regulated by Aft1 in *S. cerevisiae* and negatively regulated by Sfu1 in *C. albicans* (see Text S4). This is most easily seen by comparing the effects of a Sfu1 deletion in *C. albicans* (Figure 3B) with that of an Aft1 deletion in *S. cerevisiae* (Dataset S3). Incidentally, our results also add a new regulatory branch to the iron-acquisition model, one controlled by the transcriptional regulator *SEF1* (Figure 3). Sef1 was identified in our *C. albicans* screen as a positive regulator of iron acquisition and, although it had not been previously reported, we found a similar role for the Sef1 from *S. cerevisiae* (Dataset S3).

### Using phenotypic profiles to probe features of *C. albicans* directly applicable to medicine

As assays for specific aspects of *C. albicans* pathogenesis are developed and refined, new genetic screens can be carried out using our set of deletion strains. This strategy can provide an entry point into studying a particular problem. As an example, we consider the action of two antifungal drugs.

The primary screen included resistance and sensitivity to two antifungal agents, fluconazole and fenpropimorph, which block different steps of the ergosterol biosynthetic pathway [44],[45]. We identified 34 TRKO strains with enhanced or reduced sensitivities to these drugs, only five of which (Upc2 [46], Ndt80 [47], Crz1 [48], Tac1 [49], and Rim101 [50]) had been previously described (Figure 2B). We note an unexpected discordance between the fluconazole and fenpropimorph phenotypes in some TRKOs. In many cases resistance or sensitivity was only observed with one of the two drugs, and in a few cases resistance to one drug was accompanied by sensitivity to the other.

Of the 34 TRKOs with decreased or increased drug sensitivity, eight had high specificity scores (Figure 2B). Of these, only *UPC2* exhibited a strong defect in growth under anaerobic growth conditions (Dataset S2), a phenotype consistent with a strong defect in ergosterol biosynthesis. We predict that the other seven TRs influence resistance/sensitivity through mechanisms other than activation of ergosterol biosynthetic pathways. Four of these seven TR knockouts – *ΔΔaaf1*, *ΔΔmnl1*, *ΔΔorf19.6182*, and *ΔΔorf19.5133* – acquire *resistance* to fluconazole or fenpropimorph, a phenotype that – to our knowledge – has not been previously described in either *C. albicans* or *S. cerevisiae*. Although the mechanism of this resistance is not known, several additional observations in the literature link these TRs to drug resistance. *AAF1* is upregulated in response to the antifungal drug caspofungin [51], suggesting that this TR may serve a general role in antifungal response. *MNL1* has been shown to activate stress response genes [52]. *ORF19.6182* is similar to *S. cerevisiae PDR1*, a known master regulator of drug resistance [53]. For *ORF19.5133*, the observed high-specificity fenpropimorph resistance is the first description of this regulator.

Given that over 20% of the TRKOs screened affected resistance to either fluconazole or fenpropimorph, it seems clear that a large number of genomic targets, only a few of which have been previously described, can contribute to acquisition of resistance to these compounds. Although these antifungal agents have specific and focused mechanisms of action, we conclude that susceptibility to them can be influenced by perturbations of a surprisingly large number of transcriptional circuits. We regard these observations as a starting point for more exhaustive studies of these regulators.

### The regulatory network governing colony morphogenesis and invasive growth

A central feature of *C. albicans* is its ability to grow in three distinctive morphological forms: budding yeast, pseudohyphae, and hyphae. All three forms are found at sites of infection, and the transition appears to be closely linked to pathogenesis. On solid media, *C. albicans* exhibits a variety of colony morphologies which reflect the transitions among these three cell forms [54]. A number of transcriptional regulators of colony morphology have been identified in *C. albicans*, and a subset has been extensively studied (reviewed by Whiteway and Bachewich [26]). In screening the knockout library, we noticed that a significant fraction of the TRKOs (over 25%), including many that had not been previously characterized, exhibited distinctive colony morphology phenotypes. Because of the importance of cell morphology to *C. albicans* interaction with its human host, we paid particular attention to this phenotype and its analysis.

As colonies grow, different microenvironments are formed and the different cells of the colony respond accordingly, giving a progression of colony phenotypes over time. *C. albicans* colonies are complex structures that can be described in terms of both invasiveness and colony structure. Invasive growth – penetration into the agar surface by pseudohyphae and hyphae – was scored by examination of the colony perimeter and by observing cell retention after washing the colony from the agar surface. As colonies developed, the wild-type strain exhibited invasive growth on a variety of media. The wild-type strain also exhibited a range of colony structures, depending on the time-point and media composition. The two extremes in colony structure were “wrinkled” and “smooth”. The “wrinkled” structure was characterized by heavily ridged colonies consisting of yeast, pseudohyphal and hyphal cell types. These colonies had the consistency of rubber, likely due to extensive extracellular matrix deposition, as has been described for both *C. albicans* [55] and *S. cerevisiae* [56],[57]. As these colonies grew, the invasion into the agar described above took place. The “smooth” colony structure was characterized by dome-shaped colonies consisting primarily of yeast cells and having a paste-like consistency, likely reflecting the absence of an extensive extracellular matrix.

**Figure 4:**
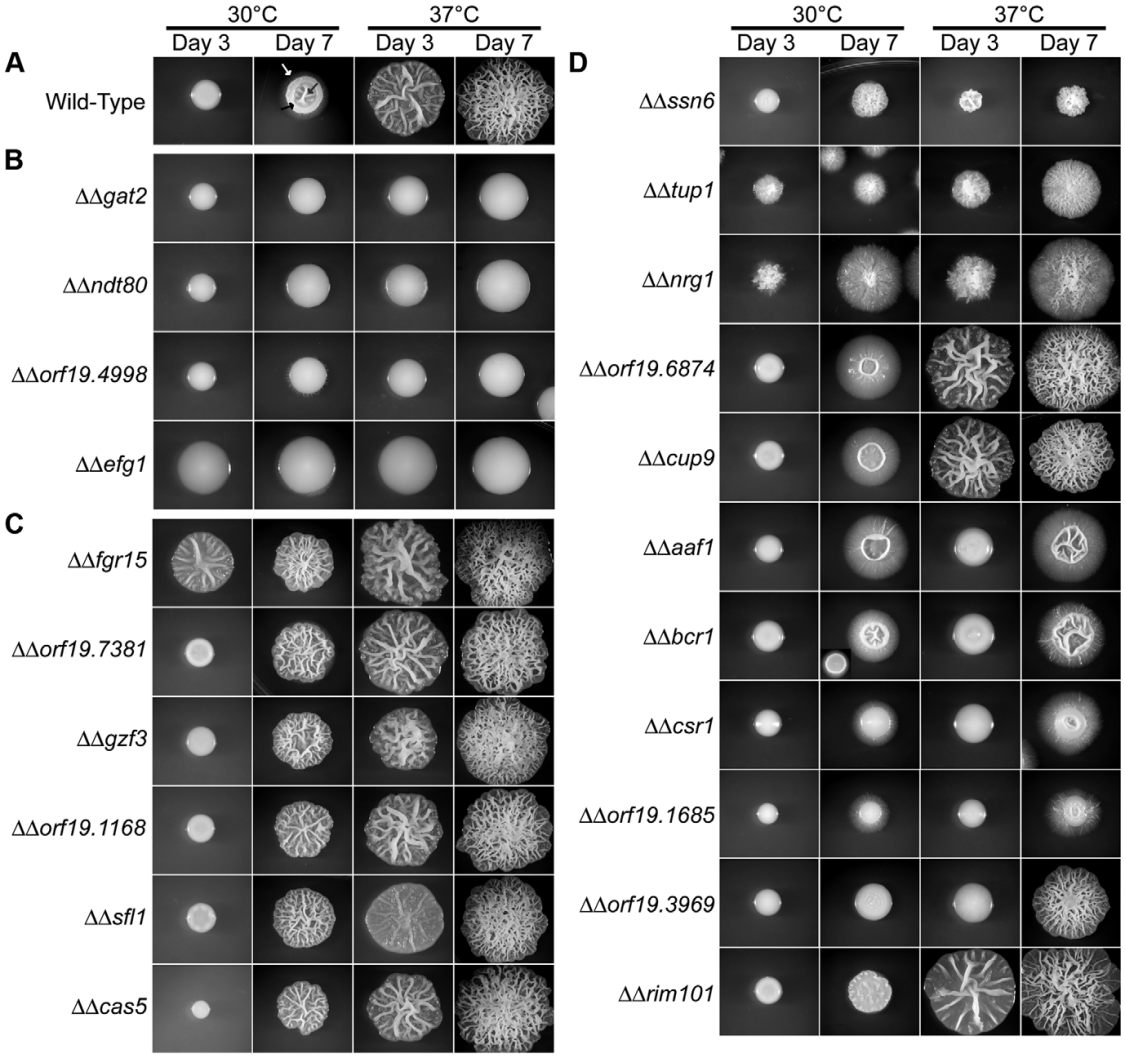
Single-cell–derived colony morphology phenotypes of *C. albicans* transcriptional regulator knockout strains. (A) Wild-type. The arrows highlight three morphological features in a single colony: peripheral invasive filaments (white arrow), a smooth colony section (black arrow), and a wrinkled colony section (grey arrow). (B) Mutants lacking both colony wrinkling and peripheral invasion. (C) Mutants with enhanced colony wrinkling on Spider at 30°C. (D) Mutants with complex morphological phenotypes. Colony morphologies of strains that exhibited a colony morphology phenotype on Spider medium in the primary screen were further analyzed using colonies grown from single cells. Colonies were photographed after 3 and 7 days of growth on Spider medium at 30°C and 37°C. Several colonies were grown on each plate, and the colony photographed on day 3 is not necessarily the same colony photographed at day 7.

The primary phenotypic screen captured the progression of colony morphology across multiple days of growth (Figure 2C), and was supplemented by a more detailed screen of colonies derived from single cells instead of patches (Figure 4). 28% of the TRKOs in our collection exhibited altered colony morphology in at least one growth condition. Although the data are extensive, several generalizations can be made. About half of the TRKOs with altered colony morphology showed a reduction in a morphological characteristic such as wrinkling or invasion, and the remaining half showed an enhancement of these features. The likely explanation, supported by a number of studies in the literature (see reviews [25],[26]), is that these morphological transitions are under both negative and positive transcriptional control. Indeed, TRs previously known to control morphology (e.g. the negative regulators of filamentous growth *NRG1* and *TUP1* and the positive regulator *TEC1*) exhibited high specificity scores. Our screen identified 20 additional transcriptional regulators that had not previously been implicated in this network.

Our results also indicate that the parameters of colony morphology can be controlled independently. For example, we observed colonies with enhanced invasion (e.g. *ΔΔorf19.6874*; see the colony periphery in Figure 4 at 30°C on day 7), colonies with wild-type levels of invasion but minimal wrinkling (e.g. *ΔΔcsr1*), colonies with enhanced wrinkling but no peripheral invasion (*e.g. ΔΔfgr15*), and colonies exhibiting neither invasion nor wrinkling (*e.g. ΔΔgat2*). Our results also indicate that some TRs can be assigned to specific features of colony development, while others act more broadly. For example, Gat2 and Orf19.4988 appear to act more generally. Deletion of either of these regulators resulted in smooth colonies with almost no invasion under all conditions tested (Figure 2C). *GAT2* has been previously identified as a positive regulator of colony morphology [58], and *ORF19.4998* is a previously uncharacterized zinc finger TR. The broad phenotypic effects of these two TRs suggest that they regulate (perhaps together) a core pathway governing the formation of colony wrinkling, extracellular matrix production, and invasion. In contrast, many other transcriptional regulators have more specific effects and are likely involved in the transmission of specific environmental signals. For example, *ΔΔorf19.1685* showed a colony morphology defect only on Spider medium, and *ΔΔorf19.2748* showed a defect only on Lee's medium (Figure 2A). The former deletion strain, *ΔΔorf19.1685*, is also deficient in the utilization of mannitol as a carbon source (Figure 2A), and mannitol is the primary carbon source of Spider medium. Similarly, the latter deletion strain (*ΔΔorf19.2748*) is unable to utilize proline as a nitrogen source (Figure 2A), and proline is highly abundant in Lee's medium. Thus these two regulators appear to link specific cues in the environment to colony phenotype.

Many other examples of TRKOs that affected colony morphology are given in Figure 2C. These results contribute to the goal of a complete description of the very large transcription circuit that controls morphological development in *C. albicans*. The results support a model in which a core pathway regulates the formation of a multi-cellular colony – consisting of different types of cells held together by an extracellular matrix – and is impinged upon by environmental cues to determine the overall output of the circuit. A next step in the analysis would be to determine, by full genome chromatin IP, the target genes for each of the core regulators. This analysis would reveal not only the transcriptional connections between the regulators themselves, but also the structural and enzymatic proteins that execute the program.

### Comparative functional analysis of orthologous *S. cerevisiae* and *C. albicans* TRs

The phenotypic analysis of the *C. albicans* TRKO collection provided an opportunity to systematically examine the conservation of transcriptional regulator function between *C. albicans* and *S. cerevisiae*. A few specific examples were discussed above, and in this section we examine the question more systematically. Specifically, we determined whether orthologous regulators in the two species controlled similar or different phenotypes. We use the term orthologous in its conventional sense, to indicate genes in the two species that derived from a single gene in the last common ancestor.

**Figure 5:**
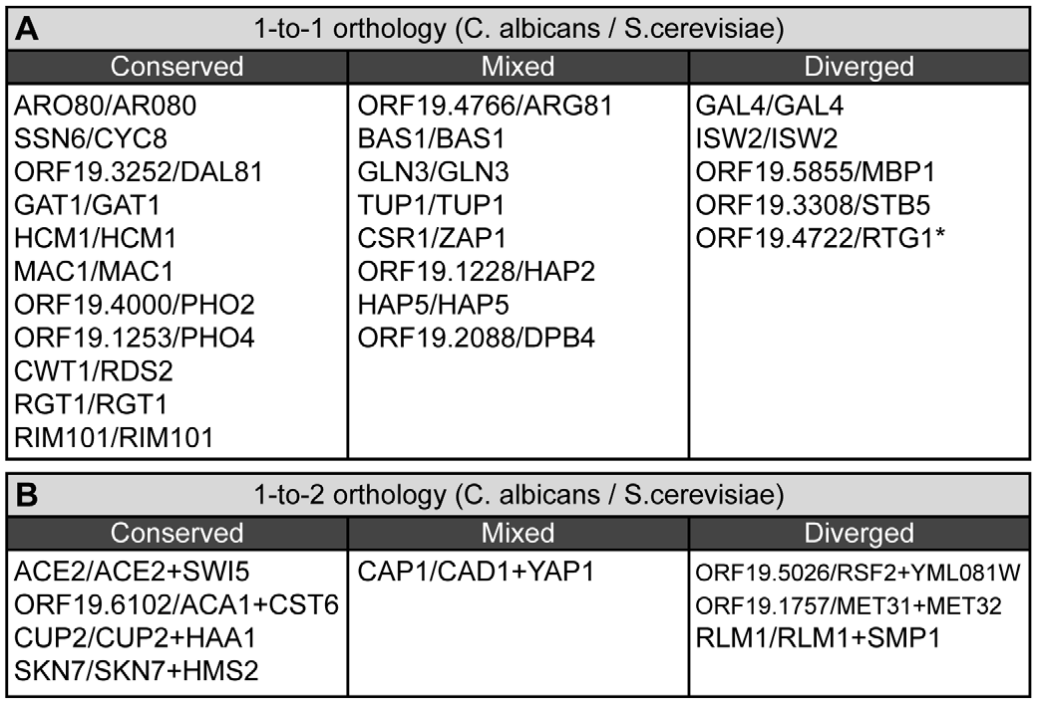
Conservation of transcriptional regulator phenotypes. (A) *C. albicans* and *S. cerevisiae* genes with 1-to-1 orthology. (B) Genes with 1-to-2 orthology where the two *S. cerevisiae* orthologs arose from a whole genome duplication event. ‘Conserved’ indicates that one or more phenotypes are shared between the indicated orthologs. ‘Mixed’ indicates that some, but not all, phenotypes were shared. ‘Diverged’ indicates that the phenotype(s) of the orthologs differed. The asterisk (*) indicates that orthology may be more complex than 1-to-1. Bold text for the 1-to-2 orthologs indicates a TR that differed in phenotype from the other two genes. Descriptions of the specific phenotypes compared are provided in the supporting materials (Text S5).

Before proceeding, we discuss several difficulties inherent to these inter-species comparisons, and how we addressed them. First, for the comparison to be valid, the phenotypic assays compared between species must employ similar conditions and methodologies. Although several high-throughput phenotypic analyses of *S. cerevisiae* have been conducted (e.g. [8],[9],[12],[14]), the extent of concordance between these studies is sufficiently low that these data are not suitable for inter-species comparison. To enable a more meaningful comparison, we conducted a limited phenotyping of *S. cerevisiae* TRKO mutants (Figure 1B, Dataset S3) using the same basic conditions that we employed for the *C. albicans* phenotyping. A second complication in phenotypic comparison is that baseline sensitivities to environmental cues (e.g. nutrient deprivation or drug exposure) may vary between species. Although these differences may have interesting explanations, they can result in false negatives, where the absence of phenotype in one species may simply reflect insufficient concentrations of the agent. To address this issue, our phenotypic assays of both yeasts tested a range of concentrations of agents such as caffeine, rapamycin, fluconazole, and fenpropimorph. As described in the supplemental materials (Text S2), this approach was used to select appropriate concentrations of agents for the screens. A third issue concerns confidence in the assignment of true orthologs given the gene duplications and losses that have occurred in the ascomycete lineage. In order to identify high-confidence orthologs (as opposed to mere homologs) in *C. albicans* and *S. cerevisiae*, we employed a combination of two different algorithms, SYNERGY [59] and INPARANOID [60] supplemented by case-by-case orthology assignments (Dataset S1; described in Text S2). For our comparison, we considered only ortholog pairs that: (1) produced a strong knockout phenotype in at least one of the two species, and (2) had been reliably assayed on the medium of interest in both species. These criteria produced a set of 24 1-to-1 orthologs for further analyses (Figure 5A).

The results show that most TRs with clear orthology between *C. albicans* and *S. cerevisiae* exhibit the same basic phenotype upon deletion. The conserved phenotypes ranged from the specific, such as impaired utilization of a nitrogen source or sensitivity to EDTA, to less defined phenotypes such as strongly impaired growth on rich medium (see Text S5 for details). Of the 24 pairs included in the analysis, we identified 11 cases of clear phenotypic conservation and an additional 8 cases where primary phenotype(s) were present in both species but where one or more additional phenotypes were exhibited by one species but not the other.

Despite the trend toward similar phenotypes produced by orthologous TRKOs, we did find exceptions, which likely reveal instances of major network rewiring. In particular, we found five cases in which a TRKO phenotype was evident in only one of the two species. One of these TRs, *GAL4*, has been previously described as a case of network rewiring [35]. *S. cerevisiae* mutants deleted for *GAL4* are unable to use galactose as a carbon source, but deletion of the *C. albicans GAL4* ortholog does not produce this phenotype ([35] and Dataset S2). A second example is seen with the regulator *RTG1*. Deletion of this regulator in *S. cerevisiae* results in glutamate and aspartate auxotrophies [61], yet deletion of the *C. albicans* ortholog does not. Although the 1-to-1 orthology between these genes is not entirely certain (a 2-to-1 relationship may exist, with *EDS1* included as a second ortholog in *S. cerevisiae*), this regulator appears to have undergone either an acquisition or loss of metabolic regulatory function since *C. albicans* and *S. cerevisiae* shared a common ancestor. It is of course possible that *C. albicans* has a redundant regulator that masks the true role of *RTG1*; however, this would still indicate that a rewiring event had occurred. Three additional differences, each suggestive of network rewiring, are listed in Figure 5A.

The data can also be used to address phenotypic conservation for orthology relationships more complex than a simple 1-to-1. Because the gene pairs that arose from the whole genome duplication (WGD) in the *S. cerevisiae* branch of the ascomycetes have been carefully curated [62], it is also possible to identify with high confidence the 1-to-2 (*C. albicans* to *S. cerevisiae*) orthologous relationships that arose from this event. Such a comparison allows us to ask whether zero, one, or both of the two *S. cerevisiae* duplicates have the same overall role as the single gene in *C. albicans*.

We analyzed eight high-confidence 1-to-2 orthologous relationships, and found several patterns of conservation (Figure 5B). First, we observed cases (exemplified by *C. albicans SKN7* and the two *S. cerevisiae* orthologs *SKN7* and *HMS2*) where the likely ancestral function was preserved in one *S. cerevisiae* gene but apparently lost in the other. Deletion of *C. albicans SKN7* and *S. cerevisiae SKN7* both result in sensitivity to oxidative stress, whereas deletion of *S. cerevisiae HMS2* does not. Thus, *HMS2* appears to have diverged (at least in the phenotypes its deletion produces) from the ancestral gene.

A second type of relationship is seen with the *S. cerevisiae MET31* and *MET32* genes relative to the single *C. albicans* ortholog, *ORF19.1757*. Deletion of either *MET31* or *MET32* from *S. cerevisiae* reveals no major phenotypes, whereas the double deletion produces a methionine auxotrophy. In our screen, deletion of *C. albicans ORF19.1757* does not produce a methionine auxotrophy or any other tested phenotype. Thus, it is likely that either the *C. albicans* gene or the *S. cerevisiae* genes retain the ancestral function and the function has changed in the other species.

A third scenario is exemplified by comparison of the *C. albicans* gene *ORF19.5026* with the two *S. cerevisiae* orthologs *YML081w* and *RSF2*. Of these three genes, only *YML081w* exhibited a phenotype (impaired growth in rich medium) under the range of conditions tested.

While far from complete, our data represent a first step towards a systematic approach to the analysis of the phenotypic output of regulatory networks in divergent species. We found an overall conservation of phenotypic output in the majority of clear 1-to-1 orthologs, but also noted several differences. Even with the small number of 1-to-2 orthologs, we observed several different phenotypic relationships, indicating that there is likely no stereotypical pattern; instead, each case must be individually explored by experiment. We are aware that the set of high-confidence orthologs is biased against orthologs that have diverged to the extent that their assignment becomes ambiguous. Nonetheless, even our high-confidence orthologs exhibit considerable divergence, and yet the phenotypic outputs are largely conserved.

Given the significant rewiring of transcriptional networks documented in the fungal lineages [4],[5],[35],[63],[64], the high degree of phenotypic conservation we observed between *S. cerevisiae* and *C. albicans* orthologs may seem unexpected. However, we know that transcriptional rewiring can take place without losing an ancestral connection between a transcriptional regulator and a process. For example, the mating circuitry between *C. albicans* and *S. cerevisiae* has undergone extensive evolutionary rewiring [5], but the same (orthologous) regulators still govern mating in both species. Likewise, the transcriptional regulator *STE12* controls the pheromone response in *S. cerevisiae* and likely also in *C. albicans*, yet the direct target genes of *STE12*, as well as the pheromone response itself, differs significantly between the two yeasts [64]–[66]. Our results indicate that despite the rewiring that has taken place, the overall function of transcriptional regulators (defined broadly by the phenotypes caused by their deletion) often remains preserved from the common ancestor.

## Materials and Methods

### Strains and media

The *C. albicans* deletion library will be made available through the Fungal Genetics Stock Center (http://www.fgsc.net/). All *C. albicans* deletion strains were constructed in strain SN152 using auxotrophic marker cassettes targeted with long-flanking homology, as previously described [23]. All deletions were verified by diagnostic PCR of the flanks surrounding the introduced markers. The absence of the gene targeted for deletion was further verified by attempting to amplify a small internal fragment of the ORF. For a successful deletion, this intra-ORF PCR yielded no product while a wild-type control yielded a strong product. The strain background of the deletion strains was *arg4Δ/arg4Δ*, *leu2Δ/leu2Δ*, *his1Δ/his1Δ*, *URA3/ura3Δ*, *IRO1/iro1Δ*, with *HIS1* and *LEU2* function restored by the auxotrophic marker introduced at the targeted transcriptional regulator. A ‘wild-type’ control strain was created by reintroduction of a single allele of *HIS1* and *LEU2* (amplified from the *C. albicans* strain SC5314) into the parent strain. Composition of the media used for phenotyping is described in Text S1.

All *S. cerevisiae* deletion strains were obtained from the Saccharomyces Genome Deletion Project collection [11]. All *S. cerevisiae* deletion strains were from the homozygous deletion collection (MATa/α *his3Δ1/his3Δ1*, *leu2Δ0/leu2Δ0*, *lys2Δ0/LYS2*, *MET15/met15Δ0*, *ura3Δ0/ura3Δ0*), with the exception of the *Δsko1* strain, which was haploid (MATa *his3Δ1*, *leu2Δ0*, *met15Δ0*, *ura3Δ0*). In all cases where the *S. cerevisiae* strain exhibited a phenotype that appeared divergent from the *C. albicans* ortholog(s), the *S. cerevisiae* strain deletion was validated using the primers suggested by the *Saccharomyces* Genome Deletion Project protocols.

### Phenotyping assays

The primary phenotypic screen assayed the growth and colony morphology of two or more independent isolates of each deletion strain on a variety of media. Strains were streaked from frozen glycerol stocks to YEPD medium and incubated overnight at 30°C. In the morning, the strains were thinly re-streaked to new YEPD plates and incubated an additional 4–6h at 30°C. This second growth period was included to ensure that the majority of cells were actively growing. Cells from each strain were then diluted in water to an OD_600_ of 0.080 (‘1×’ dilution) and transferred to a 96-well plate where an additional ‘5×’ dilution was made in the neighboring column of the plate. This format allowed the plating of 24 ‘1×’ and ‘5×’ strain dilutions with a 48-pin bolt replicator (V&P Scientific; VP 404A) to each of the assay plates. At least one wild-type control strain was included with each batch of 24 strains.

The plates were incubated and photographed over the course of a week using a Nikon CoolPix 4300 camera. The days photographed varied among the media types, depending upon the growth rate and the emergence of colony morphologies. All images were imported into custom Java viewing software for subsequent scoring and analysis. A detailed explanation of the criteria used for scoring phenotypes and the algorithm used for compiling the data is provided in Text S2. The phenotyping methodology used in the primary screen was modified in some of the follow-up phenotyping screens (see Text S2 for details).

## Supporting Information

Text S1 Composition and mechanism of action of phenotyping media.
(0.74 MB PDF)

Text S2 Supplemental materials and methods.
(1.04 MB PDF)

Text S3 Analysis of TOR pathway phenotypes.
(0.35 MB PDF)

Text S4 Analysis of copper/iron homeostasis phenotypes.
(0.29 MB PDF)

Text S5 Phenotypic comparison between *C. albicans* and *S. cerevisiae* orthologs.
(0.45 MB PDF)

Dataset S1 A curated list of *Candida albicans* transcriptional regulators.
(0.20 MB XLS)

Dataset S2 *Candida albicans* phenotype data from primary and supplemental screens.
(0.89 MB XLS)

Dataset S3 *Saccharomyces cerevisiae* phenotype data from supplemental screening.
(0.18 MB XLS)
