# Supplementary material for: A Phenotypic Profile of the Candida albicans Regulatory Network
Source: PLoS Genet. 2009 Dec 24;5(12):e1000783. doi: 10.1371/journal.pgen.1000783 (PMC2790342; doi:10.1371/journal.pgen.1000783)
Supplement: Text S1 — Composition and mechanism of action of phenotyping media. (0.74 MB PDF) [file pgen.1000783.s001.pdf]

Contents

Introduction 3

Base Media 3

YEPD [Yeast Extract Peptone Dextrose] (16°C, 30°C, 37°C, 42°C) 3

SD+Arg+Leu [Synthetic Defined + L-Arg + L-Leu] (16°C, 30°C, 37°C, 42°C) 4

MM [Minimal Medium] 4

Stress Media 5

Temperature - Low (16°C) 5

Temperature - High (42°C) 5

Copper (13mM and 15mM) 5

Lithium Chloride (300mM) 6

Zinc (1mM, 5mM, and 10mM) 6

Caffeine (15mM) 7

Rapamycin (5nM, 10nM, 15nM, 20nM, 25nM) 7

Fluconazole (100µM) 7

Fenpropimorph (2µM and 4µM) 8

Fluphenazine (0.3mM and 1mM) 8

5-FC [5-Fluorocytosine] (3.5µM) 8

Calcofluor White (20µM) 9

Sodium Dodecyl Sulfate (0.04%) 9

Sorbitol (1.5M) 9

Alkaline pH (pH10.5) 10

Acidic pH (pH2.1, pH2.6) 10

Menadione (80µM or 90µM) 10

Hydrogen Peroxide (4.5mM and 6.0mM) 11

Anaerobic Chamber 11

Nutritional Media 11

SC+uri [Synthetic Complete + uridine] 11

SC+uri-his [SC+uri - histidine] 12

SC+uri-ade [SC+uri - adenine] 12

YEPD+ade+uri [YEPD + adenine + uridine] 12

N-source: Absent 12

N-source: Proline (40mM) 12

N-source: GABA (40mM) 13

N-source: Isoleucine (15mM) 13

N-source: Ornithine (15mM) 13

YEP [Yeast Extract Peptone] 13

YEPGlycerol [Yeast Extract Peptone Glycerol] 14

C-source: Absent 14

C-source: Galactose 14
C-source: Mannitol..... 14
C-source: Maltose 15
P-source: Absent 15
EDTA [Disodium ethylenediaminetetraacetate dehydrate] (0.75mM) 15
BCS [Bathocuproinedisulfonic acid] (0.15mM) 16
BPS [Bathophenanthrolinedisulfonic acid] (0.15mM)..... 16
Morphology Media 16
Invasion - YEPD 16
Invasion - SD+Arg+Leu 17
Lee's Medium (pH4.5 and pH6.8, 30°C and 37°C)..... 17
Spider Medium (30°C and 37°C) 17
Serum (10%)..... 18
YEPD - pH7.3 18
BBL™ CHROMagar™ Candida medium[68] 18
Phloxine B (6.03µM, 25°C) 19
Blood Agar (37°C)..... 19
Blood Agar + Glucose (37°C) 20
References 21

Introduction

This supplement provides information about the media utilized in this study. The media are divided into four basic categories, with the understanding that there is considerable overlap between categories:

1. ***BASE MEDIA:*** The three types of media that were used as the base to which additional stress agents were added (e.g. fluconazole) or nutritional components were subtracted (e.g. carbon source)
2. ***STRESS MEDIA:*** Media assaying resistance and sensitivity to stress agents
3. ***NUTRITIONAL MEDIA:*** Media assaying utilization of nutrient sources
4. ***MORPHOLOGY MEDIA:*** Media inducing formation of colony wrinkling, invasive growth as well as media involving colorimetric readout.

The following categories of information are provided for each entry:

- **TITLE:** Corresponds to the name of the media utilized in all materials related to this study. When applicable, the supplement concentrations and temperatures (other than 30°C) tested in the primary screen are provided.
- **COMPOSITION:** The full recipe for the media.
- **DESCRIPTION:** Information about the mechanism of action.
- **COMMENTS:** Additional information relating to the utilization of the medium and interpretation of phenotypes
- **PHENOTYPES:** Highlights of some phenotypes associated with the medium. (This is not intended to be a comprehensive account!)

All details provided in this supplement refer to the *C. albicans* TRKO screen. The primary difference between the *C. albicans* media and the media used for *S. cerevisiae* phenotyping was that media utilizing SD+Arg+Leu as base utilized SC+uri instead (the auxotrophies differed between species). In addition, additive concentrations were recalibrated for the *S. cerevisiae* media to achieve a level of growth inhibition similar to that observed for *C. albicans*.

(Note: For brevity, 'TR' is used as an acronym for the term Transcriptional Regulator)

Base Media

YEPD [Yeast Extract Peptone Dextrose] (16°C, 30°C, 37°C, 42°C)

COMPOSITION: 1L: 20g Glucose (SIGMA G8270), 20g Peptone (BECTON DICKINSON 211677), 10g Yeast Extract (BECTON DICKINSON 212750), 18g Bacto Agar (BECTON DICKINSON 214010)

DESCRIPTION: Standard rich medium. The typical pH of our YEPD medium is ~6.3.

COMMENTS: Growth of *C. albicans* on YEPD at elevated temperature induces the formation of a wrinkled colony morphology and invasive growth. We note that our YEPD medium produced less consistent morphological phenotypes than the other morphology-inducing media tested (e.g. Spider), possibly because of subtle differences in the medium pH between batches.

PHENOTYPES: The TR knockout strain *ΔΔorf19.1253* represents an interesting case in which growth is strongly impaired on YEPD but not on SD+Arg+Leu, SC+uri or MM at 30°C. Typically, strains exhibiting poor growth in rich medium grow poorly under all conditions tested, but in this case the defect appears

to be more specific. In particular, this mutant knockout appears to be defective in copper utilization, as the addition of copper to the plates rescues the growth defect. The YEPD base medium is more basic than the SD+Arg+Leu medium, and it has been demonstrated (in *S. cerevisiae*) that copper becomes limiting in an alkaline environment[1]. In keeping with this interpretation, the *ΔΔorf19.1253* strain exhibits a growth defect on Lee's medium at pH6.8 but not pH4.5. While *ORF19.1253* has no definitive ortholog in *S. cerevisiae*, the closest BLAST match is *scPHO4*, which is known to be sensitive to alkaline pH[1] and part of the copper and phosphate regulons (*ΔΔorf19.1253* is also unable to grow on low-phosphate medium)[2]. Interestingly, *scMAC1* is also part of the copper regulon, and the TR knockout of *C. albicans MAC1* exhibits a pattern of sickness on alkaline media similar to that of *ΔΔorf19.1253* (but the copper rescue phenotype is less evident).

SD+Arg+Leu [Synthetic Defined + L-Arg + L-Leu] (16°C, 30°C, 37°C, 42°C)

Composition: 1L: 20g Glucose, 6.7g YNB w/o Amino Acids (BECTON DICKINSON 291940), 40mg L-Arg HCl, 25mg L-Leu, 18g Bacto Agar

Description: This medium is traditionally used for selection of auxotrophic markers by selective addition/omission of amino acids. The typical pH of this medium is ~5.3.

Comments: SD was chosen as base medium for many of the tested drugs because the required concentration for toxicity was often lower in this medium. A low level of L-Leu was added to the medium for historical reasons, in part because of concern over potential position-dependent variation in expression of the reintroduced *LEU2* marker in the knockout strains. For brevity, the SD+Arg+Leu medium is sometimes referred to as 'SD' in the gene phenotype annotations.

Phenotypes: Several TR knockout strains exhibited wild-type growth on YEPD but impaired growth on SD+Arg+Leu. Among the TRs conferring this phenotype were *BAS1* and *ORF19.4000* (ortholog of *scPHO2*), as well as putative members of the CCAAT-binding complex: *HAP5*, *HAP31*, and *ΔΔorf19.1228* (ortholog of *scHAP2*). While the mechanism of the CCAAT-binding TR phenotypes remains unclear, the impaired growth of *BAS1* and *ORF19.4000* knockout strains can be traced to a defect in adenine biosynthesis. The SD+Arg+Leu growth defect of these strains was rescued on SC+uri medium but not SC+uri medium in which adenine is omitted.

MM [Minimal Medium]

Composition: 1L: 20g Glucose, 5g $(NH_4)_2SO_4$, 40mg L-Arg HCl, 20mg L-Leu, 2µg Biotin, Trace Elements (as found in standard Yeast Nitrogen Base), 1g KH_2PO_4, 0.1g NaCl, 15g Bacto Agar

Description: This medium was designed for this study for analysis of limited/alternate nutrient utilization. The media is generally similar to the SD+Arg+Leu base medium, with the following modifications: (1) All vitamins typically found in Yeast Nitrogen Base are omitted, with the exception of Biotin, which is essential. (2) The salts $CaCl_2$ and $MgSO_4$, which are typically found in Yeast Nitrogen Base, are omitted. (3) A lower agar concentration was used to reduce the levels of nutrients contributed by contamination present in Bacto Agar. (4) The supplemented level of L-Leu is slightly reduced.

Phenotypes: Phenotypes observed on MM closely mirrored those observed on SD+Arg+Leu media, suggesting that none of the TRKOs significantly impacted pathways involving biosynthesis and/or

utilization of the omitted vitamins and salts (or that these ingredients were present as contaminants in the agar).

Stress Media

Temperature - Low (16°C)

COMPOSITION: YEPD base (also SD+Arg+Leu base)

DESCRIPTION: Low temperatures reduce membrane fluidity, diffusion of membrane proteins, and membrane transport[3,4]. In bacteria, cold has been demonstrated to inhibit ribosomal translation, possibly by stabilization of RNA-RNA interactions[5].

COMMENTS: Note that *C. albicans* is more cold-sensitive than *S. cerevisiae*.

PHENOTYPES: The only TR knockouts that exhibited specific cold-sensitivity (*i.e.* wild-type levels of growth at 30°C and strongly reduced growth at 16°C) were the *ΔΔgis2*, *ΔΔfgr15*, and *ΔΔcrz2* (SD+Arg+Leu only) mutants. The *ΔΔgis2* mutant also exhibited a strong sensitivity to sorbitol, possibly by a similar mechanism (see sorbitol entry).

Temperature - High (42°C)

COMPOSITION: YEPD base (also SD+Arg+Leu base)

DESCRIPTION: Increase in temperature is accompanied by changes in membrane fluidity, protein denaturation, and up-regulation of heat shock proteins. While the primary mechanisms of heat-induced toxicity are uncertain (and likely vary), evidence from studies of *S. cerevisiae* suggests that denatured proteins and protein aggregates may contribute both indirectly through loss of function and directly through inhibition of the ubiquitin-proteasome system[6].

PHENOTYPES: The only TR knockouts that exhibited specific heat-sensitivity (*i.e.* wild-type levels of growth at 30°C and strongly reduced growth at 42°C) were the *ΔΔgzf3* and *ΔΔfgr15* mutants. In both cases, impaired growth was also observed at the host environment temperature of 37°C on SD+Arg+Leu (but not YEPD) medium. However, we note that the lack of observed impairment at 37°C on YEPD may be due to the lack of an earlier time-point on this medium, which supports much faster growth than SD+Arg+Leu.

Copper (13mM and 15mM)

COMPOSITION: YEPD base + $CuSO_4 \cdot 5H_2O$ (FISHER C493)

DESCRIPTION: The cellular machinery devoted to copper homeostasis achieves a balance between the essential function of copper as a cofactor of various enzymes and the potential of copper to cause oxidative damage by generating toxic free radicals[7]. The specific mechanisms of copper toxicity are varied, and include plasma membrane permeabilization resulting from membrane lipid peroxidation[8]. Copper also serves a key role in the maintenance of iron homeostasis[7]. Thus, copper toxicity phenotypes may also reflect perturbation of iron-utilization pathways.

COMMENTS: Copper phenotypes tend to manifest as differences in viability rather than growth rate, suggesting that the primary toxic effect occurs soon after exposure. In *S. cerevisiae*, heterogeneity in copper sensitivity has been shown to track with cell-cycle-dependent variation in reactive oxygen species[9], a process that may be primarily mediated by cell-cycle-dependent variation in *SOD1* expression[10]. In our phenotyping assay, this heterogeneity increased experimental noise, and thus we focused upon the most robust phenotypes. Two copper concentrations, 13mM and 15mM, were utilized in the primary screen. The 15mM copper plates strongly reduced the viability of wild-type, and were thus primarily useful for identification of copper-resistance phenotypes. Conversely, the 13mM plates were most useful for identification of strong copper-sensitivity phenotypes. Additional copper concentrations were tested in follow-up phenotyping assays. We note that the precise concentrations of copper may not be completely accurate: the concentration of the stock solution utilized in these latter assays appears to have differed from the original stock solution utilized in the primary screen.
PHENOTYPES: One of the most copper-sensitive TRKOs was *ΔΔcup2*, an ortholog of the *S. cerevisiae CUP2/HAA1* whole-genome-duplication gene pair. Unlike *scCUP2*, *scHAA1* does not regulate metallothionein genes[11]. (Testing of the two TRKOs in *S. cerevisiae* showed that *ΔΔcup2*, but not *ΔΔhaa1* was highly copper sensitive.) Several TRKOs implicated in iron homeostasis also exhibited copper phenotypes. These included deletions of several components of the CCAAT-binding factor[12] (copper resistant) and a TRKO of *SFU1*, a repressor of iron-utilization genes[13] (copper sensitive).

Lithium Chloride (300mM)

COMPOSITION: YEPD base + LiCl (SIGMA 310468)
DESCRIPTION: In fungi, Na^+/H^+ antiporters facilitate the maintenance of ion homeostasis upon exposure to high concentrations of Na^+ and Li^+ ions[14]. The best characterized mechanism of LiCl toxicity in *S. cerevisiae* is the inhibition of the Hal2p nucleotidase by displacement of one of the two Mg^{2+} ions at the active site with Li^+. This inhibition gives rise to increased levels of pAp and may inhibit sulphotransferases and RNA processing endonucleases. In *S. cerevisiae*, calcineurin has been shown to be important for tolerance to Na^+ and Li^+ ions, presumably through reduced induction of the *scENA1* transporter[15]. In *C. albicans* lithium has been shown to inhibit filamentation in galactose-containing media[16].
PHENOTYPES: All of the TRKOs that exhibited sensitivity to LiCl were highly pleiotropic. The majority of LiCl-sensitive strains also exhibited phenotypes on Caffeine, Rapamycin, and media associated with maintenance of cell wall integrity or osmotic balance.

Zinc (1mM, 5mM, and 10mM)

COMPOSITION: YEPD base + $ZnSO_4 \cdot 7H_2O$ (SIGMA Z4750)
DESCRIPTION: Zinc is an essential cofactor for many cellular proteins. Excess zinc is toxic, and in *S. cerevisiae* has been shown to create reactive oxygen species and interfere with iron homeostasis[17].
COMMENTS: This medium was not used in the primary screen, but was used in a follow-up screen of mutants with metal homeostasis phenotypes. It is important to note that the Zinc precipitated upon

addition to the medium, and the results were thus more difficult to interpret. (Despite this precipitation, increased Zinc concentrations did exhibit higher levels of toxicity.)

PHENOTYPES: Multiple isolates were not tested because of the issue with Zinc precipitation. The two most prominent (single-replicate) phenotypes were observed at the highest concentration of Zinc: the *ΔΔgzf3* and *ΔΔorf19.1253* strains both exhibited resistance to 10mM Zinc.

Caffeine (15mM)

COMPOSITION: YEPD base + Caffeine (SIGMA C8960)

DESCRIPTION: In *S. cerevisiae*, the purine analogue caffeine (and the drug Rapamycin) interferes with the TOR (Target of Rapamycin) signaling pathway by direct inhibition of TOR complex 1[18,19]. This inhibition results in impaired response to nutrient signaling and extension of lifespan.

COMMENTS: Caffeine and Rapamycin appear to act through the same mechanism (TOR inhibition). Although Rapamycin was not included in the primary screen, subsequent follow-up testing of both drugs confirmed a very strong phenotypic correlation.

PHENOTYPES: The caffeine-resistant TRKOs included components of the CCAAT-binding complex and several TRs with roles in the regulation of nitrogen source utilization (*STP3*, *GAT1*, and *GLN3*). The caffeine-sensitive TRKOs included several TRs with enhanced invasive growth. Caffeine and TOR phenotypes are discussed at length in the manuscript and in Text S3.

Rapamycin (5nM, 10nM, 15nM, 20nM, 25nM)

COMPOSITION: YEPD base + Rapamycin (SIGMA R0395).

DESCRIPTION: Inhibitor of the TOR pathway[20] (see Caffeine description).

COMMENTS: Rapamycin was not tested in the primary screen, but was included in a follow-up assay to confirm the expected similarity between Caffeine and Rapamycin phenotypes. Our Rapamycin stock solution was a gift of the laboratory of Dr. Christine Guthrie.

PHENOTYPES: In almost every case tested, Caffeine phenotypes were recapitulated on Rapamycin medium, confirming the presumed common mechanism of action of the two drugs. Caffeine and TOR phenotypes are discussed at length in the manuscript and in Text S3.

Fluconazole (100µM)

COMPOSITION: SD+Arg+Leu base + Fluconazole (SIGMA F8929)

DESCRIPTION: Fluconazole is an azole antifungal agent that inhibits ergosterol biosynthesis by interfering with a cytochrome P450 (*ERG11* in *C. albicans*)[21,22]. Resistance in *C. albicans* is typically conferred by up-regulation of transporters controlling Fluconazole efflux and/or mutation of *ERG11*. A link between cAMP pathway signaling and susceptibility to Fluconazole (and many cell-wall perturbing compounds) has also been established[23].

COMMENTS: The phenotypic profiles for the two ergosterol biosynthesis inhibitors tested in this study, Fluconazole and Fenpropimorph, exhibited considerable overlap. However, in a few cases the phenotype of a TR knockout was either exclusive to one of the inhibitors (*e.g. ΔΔorf19.6182*) or elicited opposite phenotypes between the two inhibitors (*e.g. ΔΔswi4*).

PHENOTYPES: Among the most Fluconazole-sensitive TRKOs were several TRs with previously established sensitivities, including (1) *UPC2*, a regulator of ergosterol biosynthesis genes[24], (2) *NDT80*, a regulator of the efflux pump gene *CDR1*[25], and (3) *CRZ1*, a target of the calcineurin signaling pathway[26,27]. Among the TRs newly identified as conferring Fluconazole-sensitivity when deleted were *GZF3* and *ORF19.6182*. Several Fluconazole-resistant TRKOs were also identified, including deletions of *MNL1*, a stress-responsive TR[28], and *CRZ2*, a TR with strong resemblance to *CRZ1*. We note that a previous analysis of a *ΔΔcrz2* mutant found no Fluconazole phenotype (or perhaps even slight sensitivity?) [27]. (Fluconazole and Fenpropimorph phenotypes are also discussed at in the manuscript.)

Fenpropimorph (2µM and 4µM)

COMPOSITION: SD+Arg+Leu base + Fenpropimorph (SIGMA 36772)

DESCRIPTION: Fenpropimorph is a morpholine antifungal that inhibits ergosterol biosynthesis by inhibiting the activity of two pathway enzymes: sterol $\Delta^{8,7}$-isomerase and C_{14} sterol reductase[29,30]. Deletion of the *C. albicans* gene C_{14} sterol reductase gene, *ERG24*, enhances resistance to Fenpropimorph (and also leads to Fluconazole resistance and Fluphenazine sensitivity)[31].

COMMENTS: The phenotypic profiles for the two ergosterol biosynthesis inhibitors tested in this study, Fluconazole and Fenpropimorph, exhibited considerable overlap. However, in a few cases the phenotype of a TR knockout was either exclusive to one of the inhibitors (*e.g. ΔΔorf19.6182*) or elicited opposite phenotypes between the two inhibitors (*e.g. ΔΔswi4*). (Fluconazole and Fenpropimorph phenotypes are also discussed at in the manuscript.)

Fluphenazine (0.3mM and 1mM)

COMPOSITION: SD+Arg+Leu base + Fluphenazine (SIGMA F4765)

DESCRIPTION: Fluphenazine is utilized pharmacologically as an antipsychotic medication, and functions as a calmodulin antagonist[32,33]. The *in vivo* mechanism of activity has not been examined extensively in fungi, and it is unclear whether calmodulin-inhibition is the sole source of toxicity.

PHENOTYPES: Phenotypes were limited and generally mild. Deletion of *CRZ1*, a calcineurin-responsive TR [26,27], resulted in weak sensitivity.

5-FC [5-Fluorocytosine] (3.5µM)

COMPOSITION: SD+Arg+Leu base + 5-Fluorocytosine (SIGMA 46850)

DESCRIPTION: 5-fluorocytosine (AKA Flucytosine or 5-FC) toxicity results from deamination to 5-fluorouracil, which is then metabolized into (1) 5-fluorouridine triphosphate, which incorporates into RNA and inhibits protein synthesis, and (2) 5-fluorodeoxyuridine monophosphate, which interferes with DNA synthesis by inhibition of thymidylate synthetase[34,35]. Mutations affecting 5-FC transport or metabolism can confer resistance to the drug, as can increased pyrimidine biosynthesis. Our phenotyping data suggest that 5-FC exposure may also result in oxidative stress (see Menadione entry).

COMMENTS: Interpretation of the numerous 5-FC phenotypes observed in this study is complicated by the utilization of *LEU2* as a selective marker for creation of the knockout strains. We found that the

absence of the *LEU2* marker correlated with increased 5-FC sensitivity (*i.e.* our heterozygous deletion strains were still *ΔΔleu2* and were all highly sensitive to 5-FC). It is therefore possible that some of the 5-FC phenotypes attributed to TRKOs are actually the result of position-dependent variation in expression of the *LEU2* marker integrated during creation of the homozygous deletion strains. Addition of excess leucine did not rescue the *ΔΔleu2* 5-FC sensitivity phenotype (data not shown). We also note that different batches of 5-FC plates exhibited slightly differing levels of growth inhibition, suggesting slight variation in drug concentrations.

Calcofluor White (20µM)

COMPOSITION: YEPD base + Calcofluor White (SIGMA F3397)

DESCRIPTION: Calcofluor White is a cell wall stain with a high affinity for chitin[36]. The presumed mechanism of toxicity is perturbation of chitin synthesis and deposition. Calcofluor white is often used (along with Congo Red, SDS, and caffeine) to screen for defects in cell wall integrity pathways.

PHENOTYPES: The most highly sensitive TR knockout strain was *ΔΔrlm1*. Unlike most strains with Calcofluor White phenotypes, this knockout exhibited no phenotypes on conditions that interfere with membrane synthesis/integrity (*e.g.* Fluconazole, SDS). A *ΔΔrlm1* mutant has previously been shown[37] to be sensitive to Congo Red (which also inhibits chitin assembly) and the antifungal drug Caspofungin, an inhibitor of β-1,3-glucan synthesis[38]. Intriguingly, while these results point to a specific cell wall defect, the phenotype may be inverted in the *S. cerevisiae* ortholog. Deletion of the *RLM1* ortholog in *S. cerevisiae* confers resistance – not sensitivity – to Calcofluor White[39].

Sodium Dodecyl Sulfate (0.04%)

COMPOSITION: YEPD base + Sodium dodecyl sulfate (SIGMA L5750)

DESCRIPTION: Sodium dodecyl sulfate (AKA SDS) is a detergent that is often utilized in phenotypic screens for cell wall integrity defects. The mode of toxicity is presumably the disruption of membrane integrity.

PHENOTYPES: One of the most strongly SDS-sensitive TR knockout strains was *ΔΔorf19.5924*. This strain also exhibits strong resistance to Calcofluor White and weak sensitivity to Fluphenazine, Fenpropimorph, and copper. *ORF19.5924* is very poorly conserved among members of the ascomycete lineage and has no clear ortholog in *S. cerevisiae*.

Sorbitol (1.5M)

COMPOSITION: YEPD base + D-Sorbitol (SIGMA S1876)

DESCRIPTION: Sorbitol is added to media to increase osmolarity and can serve both to induce osmotic stress and rescue defects in osmotic balance.

PHENOTYPES: The only strong phenotype observed was the sensitivity of *ΔΔgis2* mutants to sorbitol. This mutant also exhibited impaired growth on YEPD at 16°C. A link between osmotic stress and cold stress via the HOG pathway has recently been noted in *S. cerevisiae[40]*.

Alkaline pH (pH10.5)

COMPOSITION: YEPD base + 150mM buffered glycine

DESCRIPTION: An alkaline growth environment limits the solubility of nutrients such as iron and interferes with the maintenance of a proton gradient across the plasma membrane[41]. In *C. albicans*, alkaline pH also induces morphological differentiation, favoring hyphal growth over yeast-form growth.

COMMENTS: Here we use the weak biological buffer glycine to create the alkaline media. It should be noted that alkaline-sensitivity phenotypes could in principle be attributed to the buffer rather than the alkaline stress (however see below).

PHENOTYPES: All eight of the TRKO strains that exhibited alkaline-sensitivity also exhibited some form of metal ion-related phenotype, such as sensitivity to chelator(s), lithium, and/or copper (with the possible exception of *ΔΔupc2*). These results suggest that interference with metal ion availability/homeostasis is the primary mechanism of alkaline stress (see EDTA section).

Acidic pH (pH2.1, pH2.6)

COMPOSITION: SD+Arg+Leu base + 150mM buffered glycine

DESCRIPTION: An acidic growth environment can interfere with proton gradients and damage cellular components.

COMMENTS: Multiple different pH levels were tested because of difficulties optimizing the level of viability of the wild-type strain.

PHENOTYPES: Sensitivity to acidic pH and poor growth at 16°C in SD+Arg+Leu media were correlated, perhaps due to a common mechanism involving maintenance of proton gradients. The two TRKOs with the strongest acid-sensitivity phenotypes were *ΔΔorf19.5924* and *ΔΔorf19.7017*.

Menadione (80μM or 90μM)

COMPOSITION: YEPD base + Menadione (SIGMA M5625)

DESCRIPTION: Menadione is a quinone that can generate reactive oxygen species (ROS) by redox cycling[42]. ROS lead to oxidative stress (see H_2O_2 entry). Menadione is exported from the cell following conjugation to glutathione (GSH). In *S. cerevisiae*, evidence suggests that this GSH-Menadione conjugate is itself toxic[43].

COMMENTS: Heterogeneity in the viability of isogenic cells plated to oxidative stress media may be due to cell-cycle-dependent variation in *SOD1* expression[10] (see Copper section).

PHENOTYPES: Although several TRKOs exhibited sensitivity to Menadione, the *ΔΔcap1* mutant was the only Menadione-sensitive strain that exhibited a fairly specific response (*i.e.* it did not also exhibit numerous additional phenotypes). *CAP1* has previously been identified as a regulator of the oxidative stress response in *C. albicans*[44]. The *ΔΔcap1* mutant was also sensitive to H_2O_2 and 5-FC. It is unclear whether 5-FC generates reactive oxygen species, but it is noteworthy that (1) many of the Menadione-sensitive TRKO strains were also sensitive to 5-FC, and (2) expression profiling of the response of *C. albicans* to 5-FC[45] revealed strong induction of two genes known to be involved in protection against oxidative stress in *S. cerevisiae*: *GPX2* and *TRX1*.

Hydrogen Peroxide (4.5mM and 6.0mM)

COMPOSITION: YEPD base + H_2O_2 (SIGMA H1009)

DESCRIPTION: H_2O_2 is a reactive oxygen species (ROS) that damages cellular components, including DNA, proteins, and lipids[46]. Resistance to oxidative stress is mediated by both non-enzymatic (*e.g.* glutathione, glutaredoxin, polyamine) and enzymatic (*e.g.* catalase, superoxide dismutase, glutathione reductase) mechanisms.

COMMENTS: Heterogeneity in the viability of isogenic cells plated to oxidative stress media may be due to cell-cycle-dependent variation in *SOD1* expression[10] (see Copper section).

PHENOTYPES: Several TRKOs were sensitive to both H_2O_2 and Menadione. However, as previously reported[47], deletion of *SKN7* resulted in sensitivity to H_2O_2 but not Menadione, suggesting that the response to the primary toxic species generated by Menadione is not regulated by *SKN7* (or such regulation is redundant).

Anaerobic Chamber

COMPOSITION: YEPD+ade+uri and SC+uri media incubated at 30°C in a BBL GasPak150 system with three BBL GasPak Plus envelopes (BECTON DICKINSON 271040).

DESCRIPTION: The BBL GasPak system was used to create an anaerobic environment. This environment is highly relevant to *C. albicans* fitness, as biofilms and the gastrointestinal tract are thought to be low-oxygen environments.

COMMENTS: This condition was not included in the primary screen. Instead, the full knockout library was plated directly from glycerol stock to YEPD+ade+uri and SC+uri media and incubated at 30˚C for 6 days (first replicate) and 7 days (second replicate). The chamber was then unsealed and anaerobic growth phenotypes were assessed for strains lacking growth defects on the test media in an aerobic environment.

PHENOTYPES: The only TR that exhibited a clear deficit in anaerobic growth when deleted was the ergosterol synthesis regulator *UPC2*. This phenotype has previously been described[48].

Nutritional Media

SC+uri [Synthetic Complete + uridine]

COMPOSITION: 1L: 20g Glucose, 7g YNB w/p Amino Acids (BECTON DICKINSON 291940), 45.1mg Adenine HCl, 45.1mg Uracil, 90.3mg L-Trp, 45.1mg L-His HCl, 45.1mg L-Arg HCl, 45.1mg L-Met, 67.7mg L-Tyr, 135.4mg L-Leu, 67.7mg L-Ile, 67.7mg L-Lys HCl, 108.4mg L-Phe, 334.1mg L-Val, 451.5mg L-Thr, 451.5mg L-Ser, 100mg Uridine, 20g Bacto Agar

DESCRIPTION: A synthetic complete medium with supplemented uridine.

COMMENTS: Addition of uridine to SC plates is common practice in our laboratory because it enhances growth of *ura3* auxotrophic *C. albicans* strains (the strain utilized for the screen does not have this auxotrophy). This medium was included in the phenotyping assay to determine whether strains with fitness defects on SD+Arg+Leu plates could be rescued by addition of the omitted amino acids.

PHENOTYPES: The SD+Arg+Leu growth defects of several knockout strains are rescued by SC+uri medium (see SD+Arg+Leu section).

SC+uri-his [SC+uri - histidine]

COMPOSITION: As described for SC+uri, except Histidine is omitted.

DESCRIPTION: Medium for detection of Histidine auxotrophy.

COMMENTS: This medium was only included for a small subset of strains in the primary screen.

PHENOTYPES: See SD+Arg+Leu entry for details.

SC+uri-ade [SC+uri - adenine]

COMPOSITION: As described for SC+uri, except Adenine is omitted.

DESCRIPTION: Medium for detection of Adenine auxotrophy.

COMMENTS: This medium was only included for a small subset of strains in the primary screen.

PHENOTYPES: See SD+Arg+Leu entry for details.

YEPD+ade+uri [YEPD + adenine + uridine]

COMPOSITION: 1L: 20g Glucose, 20g Bacto Peptone, 10g Yeast Extract, 100mg Uridine, 55mg Adenine, 20g Bacto Agar

DESCRIPTION: Rich medium with additional supplement of Adenine and Uridine.

COMMENTS: This medium is a variation of the conventional YEPD medium that is typically used in our laboratory. For historical reasons, this medium was used in the assay for anaerobic growth defects.

PHENOTYPES: See anaerobic medium entry.

N-source: Absent

COMPOSITION: MM base without $(NH_4)_2SO_4$

DESCRIPTION: Control plate in which the nitrogen source is omitted.

COMMENTS: Note that even without the added nitrogen source some growth is supported. Thus, this medium serves as a baseline for the 'N-source: GABA' and 'N-source: Proline' media.

PHENOTYPES: No noteworthy phenotypes were observed with this medium.

N-source: Proline (40mM)

COMPOSITION: MM base with 40mM L-Proline in place of $(NH_4)_2SO_4$

DESCRIPTION: Proline can be utilized as a non-preferred nitrogen source in *S. cerevisiae*[49].

PHENOTYPES: The only TR knockout that exhibited a strong defect in utilization of Proline as a nitrogen source was *ΔΔorf19.2748* (see Lee's medium section). Interestingly, the *ΔΔgln3* mutant strain grew slightly better on 'N-source: Proline' (and 'N-source: GABA') medium than the MM base medium. This result may be due to reduced capacity for ammonium transport, as it has recently been demonstrated that *GLN3* is an activator of the ammonium permease gene *MEP2*[50].

N-source: GABA (40mM)

Composition: MM base with 40mM Gamma-Aminobutyric acid (SIGMA A5835) in place of $(NH_4)_2SO_4$

Description: GABA, a product of glutamate decarboxylation, can be utilized as a non-preferred nitrogen source by *S. cerevisiae*[51].

Phenotypes: The TRKO *ΔΔorf19.7570* exhibited a strong growth defect on N-source: GABA medium. sc*UGA3*, a *S. cerevisiae* ortholog of *ORF19.7570*, is a known regulator of GABA catabolism[52].

N-source: Isoleucine (15mM)

Composition: MM base with 15mM L-Isoleucine in place of $(NH_4)_2SO_4$

Description: Used to assay impairment in the utilization of the amino acid Isoleucine as a nitrogen source.

Comments: *(Images from this assay were not included in the phenotype viewing software.)* This medium was not utilized in the primary screen, but was tested in a less sensitive screen in which strains were plated directly from the glycerol stock.

Phenotypes: The only TRKO to exhibit clear growth impairment on N-source: Isoleucine medium was *ΔΔaro80*. However, we note that the two *ΔΔaro80* isolates tested were not fully independently derived.

N-source: Ornithine (15mM)

Composition: MM base with 15mM Ornithine in place of $(NH_4)_2SO_4$

Description: Used to assay impairment in the utilization of the amino acid Ornithine as a nitrogen source.

Comments: *(Images from this assay were not included in the phenotype viewing software.)* This medium was not utilized in the primary screen, but was used to specifically test whether the *ΔΔorf19.4766* deletion strain shared the Ornithine-utilization defect observed upon deletion of the *S. cerevisiae* ortholog *ARG81*[53]. The assay was conducted by streaking the TRKO isolates along with the wild-type control on N-source: Ornithine and N-source: Absent medium.

Phenotypes: The *ΔΔorf19.4766* TRKO strain was defective in the utilization of Ornithine.

YEP [Yeast Extract Peptone]

Composition: 1L: 20g Peptone, 10g Yeast Extract, 18g Bacto Agar

Description: Medium designed to assay growth in the absence of a carbon source supplement.

Comments: These plates support considerable growth, presumably through the utilization of alternate carbon sources present in the yeast extract and/or peptone.

Phenotypes: The knockout strains of two TRs, *HAP5* and *ORF19.1228* (ortholog of *S. cerevisiae HAP2*), exhibited wild-type growth on YEPD but impaired growth on YEP (see CHROMagar section).

YEPGlycerol [Yeast Extract Peptone Glycerol]

COMPOSITION: 1L: 3% Glycerol, 20g Peptone, 10g Yeast Extract, 20g Bacto Agar

DESCRIPTION: A rich medium containing the non-fermentable carbon source glycerol. Unlike *S. cerevisiae*, *C. albicans* favors respiration over fermentation in the presence of oxygen[54].

COMMENTS: YEP medium supported growth rates comparable to YEPGlycerol, suggesting that the alternative carbon sources in the YEP medium are utilized preferentially over Glycerol.

PHENOTYPES: YEPGlycerol elicited the same phenotypes as YEP medium.

C-source: Absent

COMPOSITION: MM base without Glucose

DESCRIPTION: Control plate in which the carbon source is omitted.

COMMENTS: Even without glucose some growth is supported, but the level of growth is substantially less than observed for YEP medium, suggesting that either the Yeast Extract or Peptone are the primary sources of carbon in that medium. This medium serves as a baseline for the 'C-source: Galactose' medium.

PHENOTYPES: No noteworthy phenotypes were observed with this medium.

C-source: Galactose

COMPOSITION: MM base with 2% Galactose in place of Glucose

DESCRIPTION: Galactose is converted to glucose-6-phosphate and utilized as a carbon source via the Leloir pathway[55].

COMMENTS: A recent study describes a rewiring of the regulatory circuit controlling galactose metabolism in *C. albicans*[56].

PHENOTYPES: No strong phenotypes were observed with this medium.

C-source: Mannitol

COMPOSITION: MM base with 2% Mannitol in place of Glucose

DESCRIPTION: Used to assay impairment in the utilization of the polyol Mannitol as a carbon source.

COMMENTS: *(Images from this assay were not included in the phenotype viewing software.)* This medium was not utilized in the primary screen, but was utilized in conjunction with 'C-source: Maltose' medium to specifically test the capacity of *ΔΔorf19.1685* and *ΔΔsuc1* to utilize Maltose and Mannitol as carbon sources.

PHENOTYPES: The TRs *SUC1* and *ORF19.1685* genes both share similarity with several *S. cerevisiae* genes involved in maltose utilization. *SUC1* is a likely ortholog of the maltose activator *scYPR196w*, and exhibits impaired growth on 'C-source: Maltose' but not 'C-source: Mannitol' media. The deletion strain of the uncharacterized *ORF19.1685* exhibited a growth defect on Lee's medium and reduced morphology on Spider medium. Both media contain mannitol as a carbon source, suggesting that *ORF19.1685* may regulate the utilization of mannitol. Indeed, this mutant strain exhibited a reduced growth rate on the 'C-source: Mannitol' (but not the 'C-source: Maltose') medium.

C-source: Maltose

COMPOSITION: MM base with 2% Maltose in place of Glucose

DESCRIPTION: Used to assay impairment in the utilization of the disaccharide Maltose as a carbon source.

COMMENTS: *(Images from this assay were not included in the phenotype viewing software.)* This medium was not utilized in the primary screen, but was utilized in conjunction with 'C-source: Mannitol' medium to specifically test the capacity of *ΔΔorf19.1685* and *ΔΔsuc1* to utilize Maltose and Mannitol as carbon sources.

PHENOTYPES: See section on 'C-source: Mannitol'.

P-source: Absent

COMPOSITION: MM base with 0.548g/L KCl in place of K_2HPO_4

DESCRIPTION: Control plate in which the phosphate source is omitted.

COMMENTS: Even in the absence of the K_2HPO_4, some residual growth is supported.

PHENOTYPES: The ΔΔORF19.1253 knockout strain exhibited a complete absence of growth on this medium (see YEPD section).

EDTA [Disodium ethylenediaminetetraacetate dehydrate] (0.75mM)

COMPOSITION: YEPD base + 0.5M pH8.0 EDTA solution (SIGMA E5134)

DESCRIPTION: EDTA is a commonly used chelator of both di- and trivalent metal ions. EDTA is also sometimes utilized to reduce flocculation of yeasts, reportedly by chelating Ca^{2+} ions and thereby altering the interaction of lectins with the cell surface[57].

COMMENTS: EDTA was originally included in the phenotyping screen to screen for metal ion transport mutants. However, EDTA also elicited a very strong morphological response. Follow-up testing included additional EDTA concentrations and an assay of morphological growth from single-cell-derived colonies of a few select TRKOs. A link between EDTA treatment and filamentation has previously been described in an experiment in which the *C. albicans INT1* gene was expressed in *S. cerevisiae*[58]. In this system, EDTA reduced filamentation, and this effect could be alleviated by addition of Mn^{2+}. This effect was not specific to *INT1*, as a similar effect was observed upon addition of EDTA to mutant *S. cerevisiae* strains that exhibited highly polarized growth. In our study, EDTA elicited the opposite effect: it enhanced filamentation and colony morphology. (Note that while the EDTA stock solution is pH8.0, addition the EDTA had negligible effect on the pH of the media.)

PHENOTYPES: All but one of the TR knockout strains that exhibited EDTA-sensitivity also grew poorly on basic medium (YEPD pH10.5). This correlation may stem from limiting iron or copper supplies, either by chelation (EDTA) or due to an alkaline environment. Copper and iron are limiting for *S. cerevisiae* in basic media, possibly due to reduced solubility and impaired transporter function[1]. The only EDTA-sensitive TR knockout strain that was not also alkaline-sensitive was *ΔΔcsr1*. The *S. cerevisiae* ortholog of this TR, *scZAP1*, is known to be involved in zinc ion homeostasis[59].

BCS [Bathocuproinedisulfonic acid] (0.15mM)

COMPOSITION: SD+Arg+Leu base + BCS (SIGMA 146625)

DESCRIPTION: BCS is a copper chelator that is commonly added to media to limit copper availability[60]. Low copper conditions have been shown to enhance invasive growth of *C. albicans*[61].

PHENOTYPES: The only TRKO strain that was strongly sensitive to BCS was *ΔΔmac1*. This strain is also sensitive to BPS and exhibits poor growth on YEPD media. These phenotypes have also been observed in a previous study, where it was noted that addition of copper rescues the poor growth on YEPD media[61].

BPS [Bathophenanthrolinedisulfonic acid] (0.15mM)

COMPOSITION: SD+Arg+Leu base + BPS (SIGMA 11890)

DESCRIPTION: BPS is an iron chelator that is commonly added to media to limit iron availability[60].

COMMENTS: After prolonged incubation with BPS, some mutants acquired a pink/red coloration, presumably signaling formation of a ferrous/BPS complex. Follow-up assays for BPS-sensitivity included additional BPS concentrations and different base media (YEPD and SC+uri).

PHENOTYPES: The BPS-sensitivity phenotypes are described in detail in the manuscript. We note that although *ΔΔcsr1* exhibited a strong BPS phenotype, we believe this is due to chelation of zinc ions at high BPS concentrations. This hypothesis is supported by several lines of evidence: (1) *CSR1* has previously been shown to regulate zinc homeostasis[62]. (2) When a broad range of BPS concentrations were tested on the BPS-sensitive mutants in the TRKO library, the *ΔΔcsr1* mutant was atypical in that it only exhibited a phenotype at the highest concentration. Moreover, this phenotype was more severe than the phenotype observed for any of the other TRKO mutants, suggesting that the elevated BPS level had crossed a chelation threshold and was limiting the availability of zinc.

Morphology Media

Invasion - YEPD

COMPOSITION: YEPD base

DESCRIPTION: The transition of *C. albicans* from yeast to hyphal forms is believed to facilitate invasion into host tissue[63]. Invasive growth was assayed using YEPD plates grown at 30°C for two days. After this period the plates were photographed before and after thoroughly washing the cells from the surface. Any cells remaining embedded in the agar were interpreted as evidence of invasive growth.

COMMENTS: After washing, the plates were incubated at 30°C for one additional day and photographed. The plates tended to be overgrown at this stage, but this final image was sometimes useful in detecting reduced invasion phenotypes. A follow-up screen assayed invasion of a subset of TRKOs more rigorously by: (1) plating cells in a checkerboard fashion to reduce edge effects, (2) testing two temperatures: 30°C and 37°C, (3) assaying invasion by washing at several time-points, and (4) including multiple wild-type controls on each plate.

PHENOTYPES: The invasive growth screen identified enhanced invasiveness in deletions of three well-characterized regulators of filamentous growth *NRG1*, *SSN6*, and *TUP1*[64]. Several additional TR

knockout strains also exhibited strongly enhanced invasion (particularly in the SD+Arg+Leu assay) with only minor growth defects on the underlying growth media. In most cases, enhanced invasion was accompanied by enhanced colony wrinkling on other media (and vice versa). An exception to this trend was the *ΔΔndt80* strain. In *C. albicans*, the only known function of *NDT80* is regulation of the drug pump *CDR1*[25]. We find that deletion of *NDT80* results in pleiotropic phenotypes, including the unusual combination of enhanced and invasion and reduced colony wrinkling phenotypes.

Invasion - SD+Arg+Leu

COMPOSITION: SD+Arg+Leu base

DESCRIPTION: Invasive growth was assayed as described for the YEPD invasive growth test, except plates were incubated three days prior to washing rather than two.

COMMENTS: Because invasion of the wild-type strain at the time of the wash step was minimal, only phenotypes involving enhanced invasion could be reliably detected. A more detailed follow-up screen was also conducted (as described in the YEPD invasive growth entry).

PHENOTYPES: See YEPD invasive growth section.

Lee's Medium (pH4.5 and pH6.8, 30°C and 37°C)

COMPOSITION: 1L: 20g Mannitol, 5g $(NH_4)_2SO_4$, 0.2g $MgSO_4{\cdot}7H_20$, 2.5g K_2HPO_4, 5g NaCl, 0.5g L-Ala, 1.3g L-Leu, 1g L-Lys, 0.1g L-Met, 0.5g L-Phe, 0.5g L-Pro, 0.5g L-Thr, 85mg L-Arg HCl, 75mg L-Ornithine, 0.2µM $ZnSO_4{\cdot}7H_20$, 0.25µM $CuSO_4{\cdot}5H_2O$, 1µM $FeCl_3{\cdot}6H_2O$, 1mM $MgCl_2{\cdot}6H_2O$, 1mM $CaCl_2{\cdot}6H_2O$, 10mg Biotin, 20g Bacto Agar, pH with NaOH

DESCRIPTION: Lee's medium induces filamentation at elevated temperatures and pH (*e.g.* 37°C at pH6.8), while supporting predominantly yeast-form growth at lower temperatures[65].

PHENOTYPES: While the Lee's medium morphological phenotypes generally track well with the phenotypes seen on Spider and YEPD, the phenotype of *ΔΔorf19.2748* represents an interesting exception. The *ΔΔorf19.2748* mutant strain exhibits a highly diminished morphological response to Lee's medium (pH6.8 at 37°C) relative to wild-type, but no comparable phenotype on Spider medium and YEPD. This mutant also exhibits a strong growth defect on media with proline as the sole nitrogen source, suggesting that the inability to import and/or utilize proline from the Lee's medium may influence morphogenesis.

Spider Medium (30°C and 37°C)

COMPOSITION: 1L: 20g Nutrient broth (BECTON DICKINSON 234000), 20g Mannitol (SIGMA M9647), 4g K_2HPO_4, 27g Bacto Agar, pH adjusted to 7.2 with NaOH

DESCRIPTION: This rich medium strongly stimulates hyphal growth and colony wrinkling in *C. albicans*, particularly at 37°C[66].

COMMENTS: Although colony wrinkling and hyphal formation were more dramatic and rapid during growth at 37°C, these phenotypes were also evident at 30°C at day 7. However, due to an increase in edge effects at this time-point it was not considered when scoring phenotypes. Nonetheless, the slower

progression of colony morphology at 30°C made it easier to identify mutants with enhanced morphology; conversely, it was easier to identify mutants with reduced morphology at 37°C.
PHENOTYPES: All TR knockout strains that exhibited growth defects on Spider medium also exhibited growth defects on YEPD, with the exception of *ΔΔhap5* and *ΔΔorf19.1228* (see discussion of the strains in CHROMagar section). All of the ~10 TR knockout strains that exhibited enhanced colony morphology at 30°C on Spider exhibited enhanced coloration on CHROMagar medium and/or growth impairment on YEPD media (with the possible exception of *ΔΔorf19.1168*, which shows only hints of growth impairment on YEPD at 42°C). Of the ~10 TR knockout strains that exhibited reduced colony morphology on Spider at 37°C, two of the two TRs, *GAT2* and *CSR1*, had previously been identified as having filamentation phenotypes in a haploinsufficiency screen that utilized Spider media[67].

Serum (10%)

COMPOSITION: YEPD base + Fetal Bovine Serum (INVITROGEN 26140-079)
DESCRIPTION: Serum has long been used to induce filamentation in *C. albicans*, and is of particular interest because serum-induced morphological differentiation may reflect response to cues that may be present in the *C. albicans* host environment. Media containing serum has previously been utilized in a haploinsufficiency screen for filamentous growth phenotypes[67].
COMMENTS: Surprisingly, the morphological phenotypes observed on Serum medium did not differ substantially from those observed on YEPD.
PHENOTYPES: No noteworthy phenotypes were observed with this medium.

YEPD - pH7.3

COMPOSITION: YEPD buffered to pH7.3 using 100mM HEPES.
DESCRIPTION: YEPD with a slightly more alkaline pH. Presumably, the buffer also diminishes that capacity of the growing colony to influence the pH of the surrounding medium during growth.
COMMENTS: This medium was only used during follow-up testing of invasive growth. Because we suspected that subtle variations in the pH of our batches of YEPD media were causing some variation in colony morphologies, we included these buffered plates in the invasive growth assay.
PHENOTYPES: The invasive growth phenotypes on this medium were indistinguishable from those observed on YEPD medium.

BBL™ CHROMagar™ Candida medium[68]

COMPOSITION: Mix purchased from manufacturer (BECTON DICKINSON 254093)
DESCRIPTION: CHROMagar is a differential medium designed for identification of *Candida* species using a proprietary mix of chromogens. While the exact nature of the chromogens has not been disclosed, the light green colonies formed by *C. albicans* on CHROMagar may be colored by a chromophore activated by β-N-galactosaminidase activity[69].
COMMENTS: Although numerous studies have demonstrated the efficiency and reliability of *Candida* species identification using CHROMagar, here we find that CHROMagar also serves to reveal intra-

species variation in mutant phenotypes. Because CHROMagar testing was added to our phenotyping panel late in testing, we also assayed CHROMagar coloration phenotypes by plating the knockout library strains directly to CHROMagar media and observing the colony color phenotypes over the course of 13 days of growth at 30°C and 37°C. We have also observed, but not carefully characterized, colored sectors appearing in colonies grown on this media during late stages of growth.

PHENOTYPES: Three atypical colony colorations were observed:

1. DARK BLUE: Approximately 30 different knockouts developed, to varying degrees, a steel-blue color that is more typical to *C. tropicalis* than *C. albicans.* In some cases, the coloration was confined to the center of the colony (*e.g. ΔΔupc2*), and in other cases it extended into the plate agar (*e.g. ΔΔbcr1*). This dark coloration was correlated with impaired growth and enhanced invasiveness phenotypes on YEPD plates. One notable exception to this rule was *ΔΔcsr1*, a strain that grows well on YEPD and exhibits reduced morphology phenotypes on a few media types.
2. WHITE: Three knockouts, *ΔΔgat2, ΔΔmac1*, and *ΔΔorf19.4998* produced nearly white colonies, suggesting an inability to activate any chromogens. These two knockouts were among the most strongly impaired for invasive growth on YEPD, and also lacked wild-type colony morphology on a variety of media.
3. TURQUOISE: The knockout strains of two TRs, *HAP5* and *ORF19.1228* (putative ortholog of *S. cerevisiae HAP2*), produced turquoise colonies that were clearly distinct from the typical *C. albicans* light green colony color. Both mutants have virtually identical phenotypic profiles, and are thought to be components of the *C. albicans* CCAAT-binding factor[12]. Interestingly, both strains exhibited reduced growth on rich media with non-glucose sugars (YEP, YEPGalactose, YEPGlycerol, and Spider). It has previously been shown that *ΔΔHAP5* strains cannot utilize YEPLactate media[12], and that the *S. cerevisiae* CCAAT-binding factor is essential for growth on non-fermentable carbon sources[70]. Given that many chromophores utilize a sugar moiety, this carbon-utilization phenotype may be linked to the atypical colony color.

Phloxine B (6.03μM, 25°C)

COMPOSITION: Lee's Medium pH6.7 base (with 2.5% Glucose in place of Mannitol) + Phloxine B (SIGMA P4030)

DESCRIPTION: Phloxine B is a commonly used biological stain that can be added to media to differentially stain the white and opaque growth forms of *C. albicans*[71].

COMMENTS: Note that this is the only phenotyping condition that utilized a growth temperature of 25°C. Also note that the medium contains glucose rather than mannitol.

PHENOTYPES: Coloration typically took up to a week to develop, and was observed in only a small number of mutant strains, all of which exhibited some degree of growth defect on YEPD and SD+Arg+Leu media.

Blood Agar (37°C)

COMPOSITION: Pre-poured plates purchased from manufacturer (REMEL R01202)

DESCRIPTION: Blood agar plates contain TSA (trypticase soy agar) and 5% sheep blood. *C. albicans* is capable of secreting a hemolytic factor that produces a zone of clearing in the blood agar, but only when glucose is added to the plates (unlike the plates used in this study) and an incubation temperature of 37°C is utilized[72].

PHENOTYPES: No blood agar-specific growth phenotypes were observed. TRKOs that grew poorly on blood agar also grew poorly on YEPD at 37°C. One possible exception was the TR mutant strain *ΔΔorf19.6874*, which exhibited a strong invasive growth response on blood agar medium (thus making scoring of growth difficult).

Blood Agar + Glucose (37°C)

COMPOSITION: Pre-poured plates purchased from manufacturer (REMEL R01202) with an overlay of 1.875ml 40% glucose (final concentration of ~3%).

DESCRIPTION: See Blood Agar (37°C) section.

COMMENTS: These plates were not used in the high-throughput phenotyping study, but were utilized in a later screen for hemolysis defects. The TR knockout library was stamped directly from the glycerol stock to these plates, which were then incubated at 37°C. Almost all strains rapidly exhibited a zone of clearing, but the *ΔΔtye7* strain exhibited a significant (~24h) delay in the onset of hemolysis (data not shown).

References

1. Serrano R, Bernal D, Simon E, Arino J (2004) Copper and iron are the limiting factors for growth of the yeast Saccharomyces cerevisiae in an alkaline environment. J Biol Chem 279: 19698-19704.
2. Gross C, Kelleher M, Iyer VR, Brown PO, Winge DR (2000) Identification of the copper regulon in Saccharomyces cerevisiae by DNA microarrays. J Biol Chem 275: 32310-32316.
3. Schade B, Jansen G, Whiteway M, Entian KD, Thomas DY (2004) Cold adaptation in budding yeast. Mol Biol Cell 15: 5492-5502.
4. Aguilera J, Randez-Gil F, Prieto JA (2007) Cold response in Saccharomyces cerevisiae: new functions for old mechanisms. FEMS Microbiol Rev 31: 327-341.
5. Ermolenko DN, Makhatadze GI (2002) Bacterial cold-shock proteins. Cell Mol Life Sci 59: 1902-1913.
6. Riezman H (2004) Why do cells require heat shock proteins to survive heat stress? Cell Cycle 3: 61-63.
7. De Freitas J, Wintz H, Kim JH, Poynton H, Fox T, et al. (2003) Yeast, a model organism for iron and copper metabolism studies. Biometals 16: 185-197.
8. Avery SV, Howlett NG, Radice S (1996) Copper toxicity towards Saccharomyces cerevisiae: dependence on plasma membrane fatty acid composition. Appl Environ Microbiol 62: 3960-3966.
9. Howlett NG, Avery SV (1999) Flow cytometric investigation of heterogeneous copper-sensitivity in asynchronously grown Saccharomyces cerevisiae. FEMS Microbiol Lett 176: 379-386.
10. Sumner ER, Avery AM, Houghton JE, Robins RA, Avery SV (2003) Cell cycle- and age-dependent activation of Sod1p drives the formation of stress resistant cell subpopulations within clonal yeast cultures. Mol Microbiol 50: 857-870.
11. Keller G, Ray E, Brown PO, Winge DR (2001) Haa1, a protein homologous to the copper-regulated transcription factor Ace1, is a novel transcriptional activator. J Biol Chem 276: 38697-38702.
12. Johnson DC, Cano KE, Kroger EC, McNabb DS (2005) Novel regulatory function for the CCAAT-binding factor in Candida albicans. Eukaryot Cell 4: 1662-1676.
13. Lan CY, Rodarte G, Murillo LA, Jones T, Davis RW, et al. (2004) Regulatory networks affected by iron availability in Candida albicans. Mol Microbiol 53: 1451-1469.
14. Wadskog I, Adler L (2003) Ion homeostasis in Sachharomyces under NaCl stress. In: Hohman S, Mager PWH, editors. Yeast Stress Responses. Berlin/Heidelberg: Springer. pp. 201-239.
15. Mendoza I, Rubio F, Rodriguez-Navarro A, Pardo JM (1994) The protein phosphatase calcineurin is essential for NaCl tolerance of Saccharomyces cerevisiae. J Biol Chem 269: 8792-8796.
16. Martins LF, Montero-Lomeli M, Masuda CA, Fortes FS, Previato JO, et al. (2008) Lithium-mediated suppression of morphogenesis and growth in Candida albicans. FEMS Yeast Res 8: 615-621.
17. Pagani MA, Casamayor A, Serrano R, Atrian S, Arino J (2007) Disruption of iron homeostasis in Saccharomyces cerevisiae by high zinc levels: a genome-wide study. Mol Microbiol 65: 521-537.
18. Kuranda K, Leberre V, Sokol S, Palamarczyk G, Francois J (2006) Investigating the caffeine effects in the yeast Saccharomyces cerevisiae brings new insights into the connection between TOR, PKC and Ras/cAMP signalling pathways. Mol Microbiol 61: 1147-1166.
19. Wanke V, Cameroni E, Uotila A, Piccolis M, Urban J, et al. (2008) Caffeine extends yeast lifespan by targeting TORC1. Mol Microbiol 69: 277-285.
20. Crespo JL, Hall MN (2002) Elucidating TOR signaling and rapamycin action: lessons from Saccharomyces cerevisiae. Microbiol Mol Biol Rev 66: 579-591, table of contents.
21. Sanglard D (2002) Resistance of human fungal pathogens to antifungal drugs. Curr Opin Microbiol 5: 379-385.

22. Rogers KM, Pierson CA, Culbertson NT, Mo C, Sturm AM, et al. (2004) Disruption of the Candida albicans CYB5 gene results in increased azole sensitivity. Antimicrob Agents Chemother 48: 3425-3435.
23. Jung WH, Warn P, Ragni E, Popolo L, Nunn CD, et al. (2005) Deletion of PDE2, the gene encoding the high-affinity cAMP phosphodiesterase, results in changes of the cell wall and membrane in Candida albicans. Yeast 22: 285-294.
24. Silver PM, Oliver BG, White TC (2004) Role of Candida albicans transcription factor Upc2p in drug resistance and sterol metabolism. Eukaryot Cell 3: 1391-1397.
25. Chen CG, Yang YL, Shih HI, Su CL, Lo HJ (2004) CaNdt80 is involved in drug resistance in Candida albicans by regulating CDR1. Antimicrob Agents Chemother 48: 4505-4512.
26. Onyewu C, Wormley FL, Jr., Perfect JR, Heitman J (2004) The calcineurin target, Crz1, functions in azole tolerance but is not required for virulence of Candida albicans. Infect Immun 72: 7330-7333.
27. Karababa M, Valentino E, Pardini G, Coste AT, Bille J, et al. (2006) CRZ1, a target of the calcineurin pathway in Candida albicans. Mol Microbiol 59: 1429-1451.
28. Ramsdale M, Selway L, Stead D, Walker J, Yin Z, et al. (2008) MNL1 Regulates Weak Acid-induced Stress Responses of the Fungal Pathogen Candida albicans. Mol Biol Cell.
29. Lorenz RT, Parks LW (1991) Physiological effects of fenpropimorph on wild-type Saccharomyces cerevisiae and fenpropimorph-resistant mutants. Antimicrob Agents Chemother 35: 1532-1537.
30. Marcireau C, Guilloton M, Karst F (1990) In vivo effects of fenpropimorph on the yeast Saccharomyces cerevisiae and determination of the molecular basis of the antifungal property. Antimicrob Agents Chemother 34: 989-993.
31. Jia N, Arthington-Skaggs B, Lee W, Pierson CA, Lees ND, et al. (2002) Candida albicans sterol C-14 reductase, encoded by the ERG24 gene, as a potential antifungal target site. Antimicrob Agents Chemother 46: 947-957.
32. Weiss B, Levin RM (1978) Mechanism for selectively inhibiting the activation of cyclic nucleotide phosphodiesterase and adenylate cyclase by antipsychotic agents. Adv Cyclic Nucleotide Res 9: 285-303.
33. Weiss B, Prozialeck W, Cimino M, Barnette MS, Wallace TL (1980) Pharmacological regulation of calmodulin. Ann N Y Acad Sci 356: 319-345.
34. Myers CE (1981) The pharmacology of the fluoropyrimidines. Pharmacol Rev 33: 1-15.
35. Vermes A, Guchelaar HJ, Dankert J (2000) Flucytosine: a review of its pharmacology, clinical indications, pharmacokinetics, toxicity and drug interactions. J Antimicrob Chemother 46: 171-179.
36. Roncero C, Duran A (1985) Effect of Calcofluor white and Congo red on fungal cell wall morphogenesis: in vivo activation of chitin polymerization. J Bacteriol 163: 1180-1185.
37. Bruno VM, Kalachikov S, Subaran R, Nobile CJ, Kyratsous C, et al. (2006) Control of the C. albicans cell wall damage response by transcriptional regulator Cas5. PLoS Pathog 2: e21.
38. Letscher-Bru V, Herbrecht R (2003) Caspofungin: the first representative of a new antifungal class. J Antimicrob Chemother 51: 513-521.
39. Dodou E, Treisman R (1997) The Saccharomyces cerevisiae MADS-box transcription factor Rlm1 is a target for the Mpk1 mitogen-activated protein kinase pathway. Mol Cell Biol 17: 1848-1859.
40. Hayashi M, Maeda T (2006) Activation of the HOG pathway upon cold stress in Saccharomyces cerevisiae. J Biochem (Tokyo) 139: 797-803.
41. Davis D (2003) Adaptation to environmental pH in Candida albicans and its relation to pathogenesis. Curr Genet 44: 1-7.
42. Watanabe N, Dickinson DA, Liu RM, Forman HJ (2004) Quinones and glutathione metabolism. Methods Enzymol 378: 319-340.

43. Zadzinski R, Fortuniak A, Bilinski T, Grey M, Bartosz G (1998) Menadione toxicity in Saccharomyces cerevisiae cells: activation by conjugation with glutathione. Biochem Mol Biol Int 44: 747-759.
44. Wang Y, Cao YY, Jia XM, Cao YB, Gao PH, et al. (2006) Cap1p is involved in multiple pathways of oxidative stress response in Candida albicans. Free Radic Biol Med 40: 1201-1209.
45. Liu TT, Lee RE, Barker KS, Lee RE, Wei L, et al. (2005) Genome-wide expression profiling of the response to azole, polyene, echinocandin, and pyrimidine antifungal agents in Candida albicans. Antimicrob Agents Chemother 49: 2226-2236.
46. Jamieson DJ (1998) Oxidative stress responses of the yeast Saccharomyces cerevisiae. Yeast 14: 1511-1527.
47. Singh P, Chauhan N, Ghosh A, Dixon F, Calderone R (2004) SKN7 of Candida albicans: mutant construction and phenotype analysis. Infect Immun 72: 2390-2394.
48. MacPherson S, Akache B, Weber S, De Deken X, Raymond M, et al. (2005) Candida albicans zinc cluster protein Upc2p confers resistance to antifungal drugs and is an activator of ergosterol biosynthetic genes. Antimicrob Agents Chemother 49: 1745-1752.
49. Magasanik B, Kaiser CA (2002) Nitrogen regulation in Saccharomyces cerevisiae. Gene 290: 1-18.
50. Dabas N, Morschhauser J (2007) Control of ammonium permease expression and filamentous growth by the GATA transcription factors GLN3 and GAT1 in Candida albicans. Eukaryot Cell 6: 875-888.
51. Hofman-Bang J (1999) Nitrogen catabolite repression in Saccharomyces cerevisiae. Mol Biotechnol 12: 35-73.
52. Andre B (1990) The UGA3 gene regulating the GABA catabolic pathway in Saccharomyces cerevisiae codes for a putative zinc-finger protein acting on RNA amount. Mol Gen Genet 220: 269-276.
53. Dubois E, Messenguy F (1985) Isolation and characterization of the yeast ARGRII gene involved in regulating both anabolism and catabolism of arginine. Mol Gen Genet 198: 283-289.
54. Flores CL, Rodriguez C, Petit T, Gancedo C (2000) Carbohydrate and energy-yielding metabolism in non-conventional yeasts. FEMS Microbiol Rev 24: 507-529.
55. Leloir LF (1951) The enzymatic transformation of uridine diphosphate glucose into a galactose derivative. Arch Biochem 33: 186-190.
56. Martchenko M, Levitin A, Hogues H, Nantel A, Whiteway M (2007) Transcriptional rewiring of fungal galactose-metabolism circuitry. Curr Biol 17: 1007-1013.
57. el-Behhari M, Ekome JN, Coulon J, Pucci B, Bonaly R (1998) Comparative extraction procedures for a galactose-specific lectin involved in flocculation of Kluyveromyces lactis strains. Appl Microbiol Biotechnol 49: 16-23.
58. Asleson CM, Asleson JC, Malandra E, Johnston S, Berman J (2000) Filamentous growth of Saccharomyces cerevisiae is regulated by manganese. Fungal Genet Biol 30: 155-162.
59. Wang Z, Feng LS, Matskevich V, Venkataraman K, Parasuram P, et al. (2006) Solution structure of a Zap1 zinc-responsive domain provides insights into metalloregulatory transcriptional repression in Saccharomyces cerevisiae. J Mol Biol 357: 1167-1183.
60. Hammacott JE, Williams PH, Cashmore AM (2000) Candida albicans CFL1 encodes a functional ferric reductase activity that can rescue a Saccharomyces cerevisiae fre1 mutant. Microbiology 146 (Pt 4): 869-876.
61. Marvin ME, Mason RP, Cashmore AM (2004) The CaCTR1 gene is required for high-affinity iron uptake and is transcriptionally controlled by a copper-sensing transactivator encoded by CaMAC1. Microbiology 150: 2197-2208.
62. Kim MJ, Kil M, Jung JH, Kim J (2008) Roles of Zinc-responsive transcription factor Csr1 in filamentous growth of the pathogenic Yeast Candida albicans. J Microbiol Biotechnol 18: 242-247.
63. Gow NA, Brown AJ, Odds FC (2002) Fungal morphogenesis and host invasion. Curr Opin Microbiol 5: 366-371.

64. Garcia-Sanchez S, Mavor AL, Russell CL, Argimon S, Dennison P, et al. (2005) Global roles of Ssn6 in Tup1- and Nrg1-dependent gene regulation in the fungal pathogen, Candida albicans. Mol Biol Cell 16: 2913-2925.
65. Lee KL, Buckley HR, Campbell CC (1975) An amino acid liquid synthetic medium for the development of mycelial and yeast forms of Candida Albicans. Sabouraudia 13: 148-153.
66. Liu H, Kohler J, Fink GR (1994) Suppression of hyphal formation in Candida albicans by mutation of a STE12 homolog. Science 266: 1723-1726.
67. Uhl MA, Biery M, Craig N, Johnson AD (2003) Haploinsufficiency-based large-scale forward genetic analysis of filamentous growth in the diploid human fungal pathogen C.albicans. Embo J 22: 2668-2678.
68. Odds FC, Bernaerts R (1994) CHROMagar Candida, a new differential isolation medium for presumptive identification of clinically important Candida species. J Clin Microbiol 32: 1923-1929.
69. Beighton D, Ludford R, Clark DT, Brailsford SR, Pankhurst CL, et al. (1995) Use of CHROMagar Candida medium for isolation of yeasts from dental samples. J Clin Microbiol 33: 3025-3027.
70. Brakhage AA, Andrianopoulos A, Kato M, Steidl S, Davis MA, et al. (1999) HAP-Like CCAAT-binding complexes in filamentous fungi: implications for biotechnology. Fungal Genet Biol 27: 243-252.
71. Anderson JM, Soll DR (1987) Unique phenotype of opaque cells in the white-opaque transition of Candida albicans. J Bacteriol 169: 5579-5588.
72. Manns JM, Mosser DM, Buckley HR (1994) Production of a hemolytic factor by Candida albicans. Infect Immun 62: 5154-5156.
