# Supplementary material for: A Phenotypic Profile of the Candida albicans Regulatory Network
Source: PLoS Genet. 2009 Dec 24;5(12):e1000783. doi: 10.1371/journal.pgen.1000783 (PMC2790342; doi:10.1371/journal.pgen.1000783)
Supplement: Text S2 — Supplemental materials and methods. (1.04 MB PDF) [file pgen.1000783.s002.pdf]

Contents

Phenotype Scoring: Scale and Criteria 2

Scoring scale 2

Factors influencing scoring 3

Phenotype Scoring: Score Analysis 3

1. Filter data 3

2. Collapse data to single value 4

3. Flag scores with additional descriptors 4

Phenotype Scoring: Specificity Score 5

Specificity Score: Overview 5

Specificity Score: Interpretation 6

Specificity Score: Calculation 6

Specificity Score: Data used for Calculation 7

Estimation of Pleiotropy 7

Phenotype Scoring: Merging Scores for Multiple Conditions 9

Phenotype Scoring: Assay Limitations 9

Supplemental Phenotyping Protocols 11

1. Invasive Growth (primary screen and supplemental) 11

2. Single-Cell-Colony Morphologies 12

3. Straight-from-Stock (low sensitivity assays) 12

4. Targeted Supplemental Phenotyping 12

5. S. cerevisiae TRKO phenotyping screen 13

Phenotype Validation 13

1. Reproducibility Of Additional Replicates 13

2. Overlapping Phenotypic Profiles of TRKOs Associated With Protein Complexes 13

3. Reproducibility of Previously Reported Phenotypes 13

Orthology: Classification of Orthologs 13

Orthology: Comparative Phenotype Analysis 14

References 16

Phenotype Scoring: Scale and Criteria

Scoring scale

Upon import into the viewing software, each plate photo was split into separate images for each strain on the plate. This process created over 100,000 images, all of which were assigned a growth score and a morphology score by hand using the viewing software. The scoring scale used was as follows:

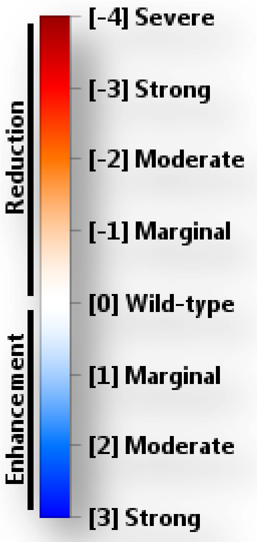

Below, we define the criteria used as they relate to the different scoring terms and to the two types of phenotype scores (growth and morphology). All comparative terms are in reference to the wild-type control.

	Growth	*Morphology**
Enhancement	Increased growth	Increased colony fuzziness and/or peripheral filamentation
Reduction	Reduced growth	Reduced colony fuzziness and/or peripheral filamentation
Severe	No growth evident for deletion strain through day 4 and at least some growth for wild-type strain.	*N/A*
Strong	Very strong difference	
Moderate	Clear but mild difference	
Marginal	*Possible* slight difference	
Wild-Type	No difference	

*For the assays scoring invasive growth and CHROMagar coloration, the morphology score was adapted to reflect the extent of retention after washing the agar and the darkness of coloration relative to wild-type, respectively.

Two additional scores were applied under special circumstances. A morphology score of '*Unusual*' was applied when the simple scoring scheme did not adequately describe the colony morphology (*e.g.* a

deletion strain with reduced fuzziness and enhanced peripheral filamentation). A score of '*Bad*' was applied to any image that could not be scored (*e.g.* due to contamination or plating error).

Factors influencing scoring

When applying the scoring criteria outlined above, three key modifying factors were taken into account:

1. Edge Effects

Edge effects were evidenced by the enhanced growth (or filamentation) of strains on the periphery of the plate. These effects became evident during later time-points, and were taken into account when judging phenotype relative to wild-type. To further reduce the influence of edge effects, images obtained after day 4 were not included in the final scoring analysis of the primary screen. While necessary for quality control, this restriction resulted in the omission of some reproducible morphological phenotypes that only manifested at later time-points. We refer the reader to the viewing software to further explore these phenotypes.

2. Medium Reliability

While subtle differences in batches of media (e.g. slight variations in drug concentration) were accounted for by the inclusion of a wild-type control on each plate, this control was insufficient in cases where intra-plate variability was observed. We assumed that a strong majority of strains on any given plate would resemble the wild-type strain and substantially increased the threshold for calling a phenotype when this was not the case. This was only necessary for media that was bordering on lethal to wild-type, such as SDS, our highest copper concentrations, and our low pH conditions. These conditions also differed from most of our other test conditions in that they influenced viability far more dramatically than growth rate. The latter effect is far easier to score in our assay.

3. Phenotypic Range

Scoring of the severity of a phenotype was scaled to reflect the phenotypic range of all observed phenotypes. This adjustment was only applied when the condition tested yielded highly reproducible results and there was *a priori* reason to expect that the range of phenotypes would be small. The primary application of this adjustment was the scoring of nutritional phenotypes (*e.g.* low phosphate, low iron) where some basal growth would still occur even in the case of a strong defect.

Phenotype Scoring: Score Analysis

After all data from the primary screen were compiled and scored, the scores were processed as follows:

1. Filter data

Several categories of data where omitted from the final analysis:

- Only the first four days of growth were considered for analysis of the primary screen (see above).
- Some data were omitted from the final phenotyping because they were clearly anomalous. The primary example is the omission of data utilizing our first batch of Lee's medium and Spider medium, as subsequent testing revealed that the growth rate and morphological response to these media was highly atypical.
- All images flagged as *'Bad'* during scoring were also omitted from analysis.
- In cases where more than two independent strain isolates were assayed (typically to resolve phenotypic inconsistencies), if one isolate was a clear outlier it was omitted.

2. Collapse data to single value

For each deletion strain and phenotyping condition all available growth scores were distilled to a single scoring value as follows:

Symbols	
S_d	Phenotype score for the day, 'd'
d	Day of photograph
i	TRKO isolate
I	The set of all isolates for a given TRKO
r	Phenotyping replicate
R_i	The set of all phenotyping replicates for a given isolate 'i'
S_{ir}	Phenotype score for isolate 'i' and replicate 'r'

1. For each TRKO, a score (S) for each photographed day (*d*) was obtained by first averaging intra-isolate replicates and then taking the inter-isolate average:

$$S_d = \frac{1}{|I|}\sum_{i \in I}\left(\frac{1}{|R_i|}\sum_{r \in R_i} S_{ir}\right)$$

(Note that $|I|$ and $|R_i|$ refer to cardinality of the set.)

2. Because no scores beyond day 4 were utilized and at least two days of growth were photographed for each medium, this process yielded between two and four scores for each TRKO and medium. From these scores, the score with the highest absolute value was chosen as the final score. (Note that it is formally possible for a strain to have reduced growth on some days and enhanced growth on others, but this did not occur in our data set.)

Morphology scores were calculated in similar fashion, with scores of '*Unusual*' being ignored (and flagged as described below).

3. Flag scores with additional descriptors

The following additional 'flags' were appended to phenotype scores based on the criteria described below:

[X]	*Could not be interpreted due to reduced growth on base medium*
[I]	*Inconsistent results*
[S]	*Only a single isolate was tested*
[P]	*Only had data for a single isolate on at least one of the photographed days*
[U]	*Unusual morphology that defied simple enhancement/reduction classification*

The criteria used for flagging growth scores were:

- **[X] → Impaired growth on base medium**: In order to prevent the false attribution of sensitivity to a phenotyping condition when the strain also exhibited impaired growth on the base medium, these instances were flagged. (The base medium used for each condition is described in Text S1) For example, YEPD at 30°C served as base medium for conditions such as YEPD at 42°C and YEPD + Caffeine. In these cases the Growth Score on the base media was compared to the Growth Score on the designated Base Medium. The criteria used for flagging were as follows:

  [X] = (Base Score ≤ -1.5) AND (Growth Score ≤ -1) AND (|(Base Score) – (Growth Score)|) < 2)

- **[S] or [P#] → Lacks multiple isolates:** In some instances a knockout strain lacked images for multiple isolates on one or more of the days scored. In cases where all days scored lacked a second isolate (typically because no second isolate existed), the **[S]** flag was appended to the score. In cases where only a subset of the days scored contained images for multiple isolates (typically for technical reasons), the **[P#]** flag was appended to the score, with the '**#**' being equivalent to the number of days for which multiple isolates were scored. An exception was made when at least one day contained images for multiple isolates and all scores for that day were either ≤ -2 or ≥ 2. (Additionally, the number of isolates scored had to equal the maximum number scored on any day.) In these cases the **[P#]** flag was omitted.

- **[I] → Inconsistencies:** A score was marked as inconsistent if the maximum difference between isolates (step#2 of scoring) exceeded a value of 1. As with the **[P#]** flag, an exception was made when all scores for at least one day were either ≤ -2 or ≥ 2.

The criteria used for flagging morphology scores were:

- **[X] → Impaired growth:** In instances where the Growth Score of the strain was ≤ -3 the Morphology Score was difficult to interpret. These scores are marked with the **[X]** flag.

- **[S] or [P#] → Lacks multiple isolates:** This flag was applied using the same criteria described for growth scoring (using Morphology Scores instead of Growth Scores).

- **[I] → Inconsistencies:** This flag was applied using the same criteria described for growth scoring (using Morphology Scores instead of Growth Scores).

- **[U] → Unusual morphology:** If any of the images were scored *'Unusual'*, this flag was appended to the score.

Phenotype Scoring: Specificity Score

Specificity Score: Overview

The specificity score, as expressed by circle diameter in Figure 2 of the manuscript, is a representation of the total number of phenotypes of a TRKO relative to the other TRKOs that also exhibited strong phenotypes on the same condition. When a TRKO had few phenotypes relative to the other TRKOs, it was assigned a high specificity score (Figure 2; large diameter). Conversely, a TRKO with a large number of phenotypes relative to the other TRKOs was assigned a low specificity score (Figure 2; small diameter).

The underlying premise behind this calculation is that the extent of pleiotropy of a TRKO is inversely correlated with the specificity (and possibly the directness) of the connection between the TR and the gene modules relevant to fitness on the medium of interest. In other words, we assume that pleiotropic TRs are more likely to act as global regulators of many processes and/or as indirect regulators that act through other more specific TRs.

We would like to stress that the specificity score is not intended as a precise mathematical description of network connectivity, but rather as a conceptual framework for considering the phenotypic data and generating hypotheses regarding which regulators are central to a given regulatory network. However, as phenotyping data becomes more abundant, we consider an expansion of the approach described below as a possible means for incorporating phenotypic output into network modeling. (Key to such an effort would be the training of models on well-defined networks to calibrate parameters such as the relative influence of pleiotropy and phenotype strength on connectivity scoring.)

Specificity Score: Interpretation

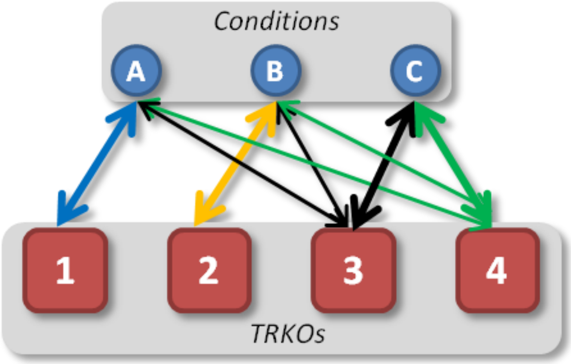

Consider a network of strong enhancement phenotypes (lines) between three conditions (A,B,C) and four TRKOs (1,2,3,4), as diagrammed above. TRKOs #3 and #4 are pleiotropic, whereas TRKO#1 and TRKO#2 are each connected to only a single phenotype. The thick lines indicate connections with high specificity scores.

The scoring for *Condition A* would be interpreted as follows:

- ***SPECIFICITY SCORE***: The connection between *TRKO#1* and *Condition A* (blue line) is assigned a high specificity score (thick line) because the TRKO is associated with only a single condition and the other TRKOs connected to the condition are more pleiotropic (they are given low specificity scores).
- ***INTERPRETATION:*** *TRKO#1* regulates the circuit relevant to *Condition A* and no other circuits. In contrast, *TRKO#3* and *TRKO#4* influence more circuits. These TRKOs may directly regulate circuits associated with multiple phenotyping conditions, or they may exert influence on *Condition A* only through *TRKO#1*.

The scoring for *Condition C* would be interpreted as follows:

- ***SPECIFICITY SCORE***: Although both TRKOs (*#3* and *#4*) that are connected to *Condition C* are pleiotropic, they are both given high specificity scores, because no TRKO with fewer phenotypes is associated with *Condition C*.
- ***INTERPRETATION:*** Although *TRKO#3* and *TRKO#4* regulate multiple circuits, they are the most direct regulators of the circuit probed by *Condition C* that were identified in the phenotypic screen. This may be due to either (1) direct regulation of the circuit relevant to *Condition C,* (2) indirect regulation of *Condition C* through TRs that were not included in our screen, or (3) due to the *Condition C* phenotype requiring activation of the circuits controlling both *Condition A* and *Condition B*.

Specificity Score: Calculation

For each phenotype condition, we calculated the specificity scores separately for the set of TRKOs that exhibited an enhancement phenotype and those that exhibited a reduction phenotype. These sets were considered separately in order to highlight the key regulators of both types of phenotype. In order to emphasize only strong phenotypes, all phenotypes with marginal or moderate scores ($|\text{score}| < 2.5$) were assigned the minimum specificity score. The specificity score for each strong phenotype was calculated as follows:

1. The estimated number of phenotypes (N) was calculated for each TRKO (see section below).
2. For each phenotype condition, we identified the lowest N among the set of TRKOs that exhibited moderate or stronger phenotypes under that condition. This calculation was made separately for enhancement and reduction phenotypes (yielding two N_{min} scores).

3. For each strong phenotype, a relative pleiotropy score (R) was calculated as the ratio of the estimated number of phenotypes (N) and the minimum estimated number of phenotypes (N_{min}) for TRKOs that exhibited a similar phenotype (i.e. enhancement or reduction) under that condition. Because the estimated number of phenotypes could actually be a small fraction (see section below), we set the minimum N_{min} value as 1.5. The choice of this value also served to reduce the distinction between TRKOs with low levels of pleiotropy. This adjustment of the N_{min} created the possibility of ratios below 1; these were reset to a value of 1. These calculations are summarized by the following formula:

$$R = max\left(1, \left(\frac{N}{max(1.5, N_{min})}\right)\right)$$

4. Because it was more intuitive for a large number to correspond to a higher specificity, the specificity score was then obtained by transforming the relative pleiotropy score:

$$\text{Specificity Score} = \frac{1}{R}$$

   Thus, the maximum specificity score is 1.00 and the minimum specificity score is 0.00.
5. The circle diameters used in Figure 2 were scaled such that the minimum diameter corresponded to a specificity score ≤ 0.25 and the largest diameter corresponded to a specificity score of 1.00. As described earlier, marginal and moderate phenotypes were also assigned the minimum diameter.

Specificity Score: Data used for Calculation

Only the phenotyping data from the primary dataset that are provided in Data Set S2 were used for calculating the specificity score. We did not utilize data from the supplemental screens for this purpose because: (1) only a small subset of the full TRKO collection was typically used for these screens, and (2) these TRKOs were selected non-randomly. However, we made two exceptions to this rule. First, we replaced the two BPS scores for *ΔΔhap5* and *ΔΔhap2* with those obtained in the follow-up screen (both values are shown in Data Set S2). This was done because in the primary screen the BPS plates utilized a base medium (SD+Arg+Leu) that did not support robust growth of these mutants, and the follow-up phenotyping revealed a clear phenotype when YEPD base was used. The second exception was the substitution of invasive growth scores from the primary screen with those from the more rigorous supplemental screen (30°C plates only; screen described below). Replaced scores were also used to determine circle color in Figure 2 of the manuscript.

Estimation of Pleiotropy

In order to assess phenotype specificity, it was necessary to calculate the number of phenotypes attributed to each TRKO. This was not a simple matter of adding up the number of observed phenotypes in our data set because of two issues:

1. Some conditions probed overlapping regulatory circuits and thus phenotypes attributed to these conditions were not independent.
2. Some TRKOs had phenotype data for only a subset of conditions (e.g. TRKOs with poor growth on base medium or score inconsistencies).

Therefore, we devised a technique that incorporated hierarchical clustering to identify closely related conditions. For each TRKO, missing data were interpolated from existing data, and then the phenotyping data were mapped onto the leaves of the tree resulting from the clustering operation.

The procedure utilized to calculate the estimated number of phenotypes is diagrammed and discussed in greater detail below.

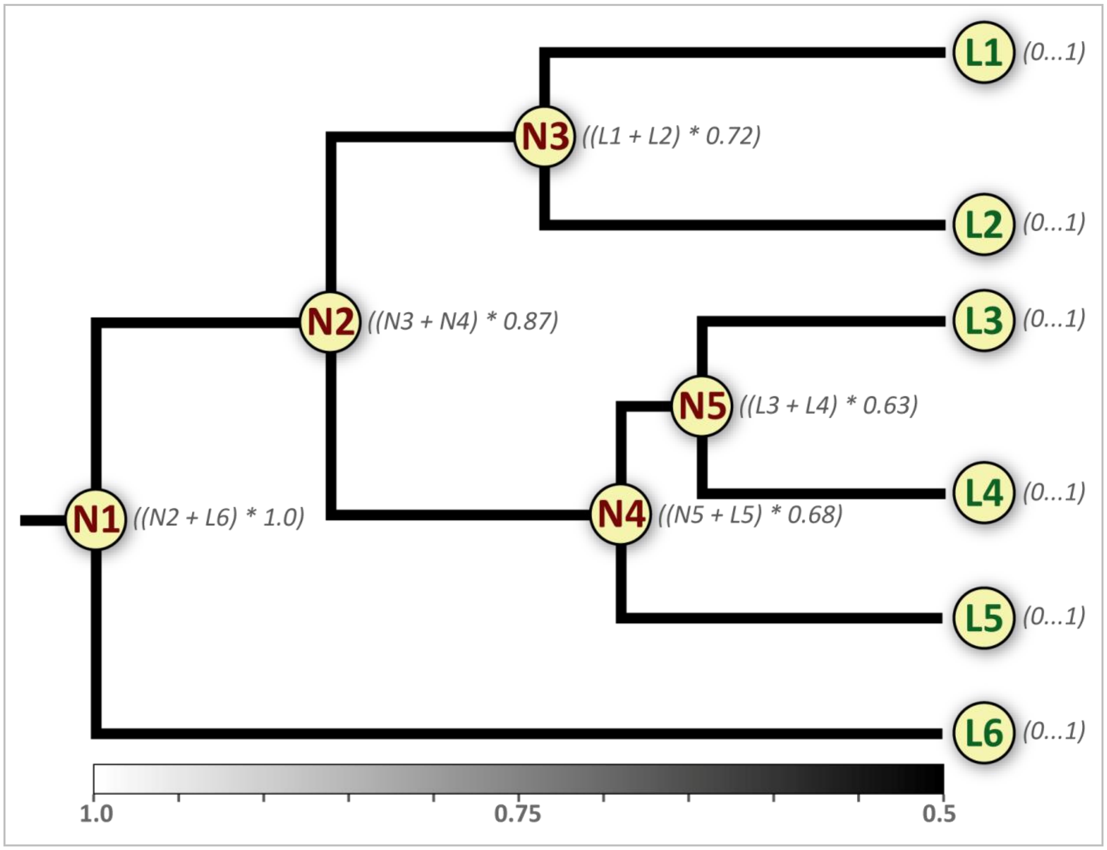

1. All phenotype scores were reduced to a simplified score of '*1*', '*0*', or '*No Value*' based on the following criteria:

   - *1* → Absolute value of the score ≥ 1.5
   - *0* → Absolute value of the score < 1.5
   - *No value* → Score flagged [X] or not available

   For the purposes of deriving the tree, all flags except '*[X]*' were ignored. The '*[X]*' flag was preserved in order to prevent clustering of phenotypes attributable to the underlying base medium.
2. The tree was derived using the software '***Cluster 3.0***' (http://bonsai.ims.u-tokyo.ac.jp/~mdehoon/software/cluster/manual/index.html)[1]. The scores were imported into '***Cluster 3.0***' as a matrix of TRKOs and phenotype conditions and clustered using the '*Complete linkage*' clustering method and the '*Correlation (uncentered)*' similarity metric.
3. For each TRKO, phenotype scores were mapped onto the leaves of the tree. The scores were derived as described for step#1, with the exception that any case of '*No value*' was replaced with the a value equal to the frequency of phenotypes for that condition among all TRKOs (*e.g.* if 15% of all TRKOs with valid scores exhibited a Fluconazole phenotype, all TRKOs missing a value for that condition would be given a score of '*0.15*'). Although this approach provided a reasonable means of handling missing data, it is important to note that the accuracy of TRKO pleiotropy estimates is reduced for TRKOs with very few data points (e.g. TRKOs that grew poorly on the base media).

4. The estimated number of phenotypes was then derived by traversing the tree from leaf to root while summing the phenotypes at each node. Each summation operation was weighted by the distance of the node from the root (see scale at bottom of figure). The weighting scale was calibrated so that summing two phenotypes from perfectly correlated conditions would yield only one total phenotype while summing two phenotypes from completely uncorrelated conditions would yield two total phenotypes.

Phenotype Scoring: Merging Scores for Multiple Conditions

In Figure 2 of the manuscript we sometimes merged multiple conditions into a single column for clarity. In these cases, the highest specificity score and the strongest phenotype score among the conditions merged were used. Below, we provide a list of the column headers and the conditions merged:

	Column Header	*Conditions Merged*
Figure 2B	*Fluphenazine*	0.3mM and 1mM
	Fenpropimorph	2uM and 4uM
	Menadione	80uM and 90uM
	H2O2	4.5mM and 6mM
	Copper	13mM and 15mM
	Acidic	pH2.1 and pH2.6
Figure 2C	*Invasion*	YEPD base and SD+Arg+Leu base

We also note that a small number of invasive growth and BPS-sensitivity scores were replaced with data from the supplemental screens (as described in the section titled *'Specificity Score: Data used for Calculation'*).

Phenotype Scoring: Assay Limitations

While our assay was generally quite robust and reproducible, we highlight some general issues that arise when scoring phenotype based on images of strains grown on solid media:

1. False positives resulting from subtle growth defects on the base medium

Typically, when a growth defect is evident on the underlying base medium all other media utilizing this base are flagged with '[X]' (see above). However, if the defect is subtle or undetected, other conditions that utilize the same base medium (*e.g.* YEPD + Caffeine) might expose the defect, leading to false attribution of the condition as the underlying cause. The most likely scenario under which this issue arises is the presence of a subtle defect that is only visible on the base media before the first time-point (typically 24h) but is visible for stress conditions using the base medium because overall growth is slowed. Warning signs of this issue are a growth score on the base medium of ~-1.0 and/or the detection of moderate or marginal growth defects for multiple stress conditions utilizing the base medium.

2. Strains that were difficult to score

Because of atypical growth morphologies, a few deletion strains were very difficult to score. These difficulties are noted in the phenotype commentaries for these strains, and are summarized here:

- ***ace2ΔΔ*:** Due to the cell-separation defect of the mutant, the resulting irregular colony growth impaired scoring of both growth and morphology.
- ***tup1ΔΔ* and *nrg1ΔΔ*:** The extremely strong filamentation and invasion phenotypes of these mutants impaired all aspects of phenotyping, including the growth and dilution of strains prior to plating.
- ***swi4ΔΔ*:** This mutant appeared to exhibit reduced viability upon plating, as the number of colonies observed in the dilution series was often less than that of other strains (alternatively, these mutants may not be amenable to density-normalization by OD). The reduced viability was difficult to score consistently, as the phenotyping growth score is better suited for scoring growth rate.
- ***efg1ΔΔ*:** The typical colony size of these mutants exceeded that of the wild-type strain, complicating the scoring of growth phenotypes.

3. Limitations to morphology scoring

'Colony morphology' is a broad term, so compromises were necessary to reduce this complex phenomenon to a single score. For more detailed analysis, we refer the reader to the more refined single-colony-derived morphology assay (described in the section below titled 'Supplemental Phenotyping Protocols'). For our primary screen, we note several issues that complicated growth scoring:

- ***Slow-growing strains*:** While strains with severe growth phenotypes were flagged (see above), weak growth defects sometimes resulted in a delay in the development of colony fuzziness. Such a delay may not be indicative of a direct morphological defect, but rather may reflect a delay in the development of the micro-environment that eventually leads to colony fuzziness. We chose not to flag these morphology scores because this trend was not universal. In some cases a strain exhibited a growth defect and yet exhibited enhanced fuzziness.
- ***Visual identification of invasive growth:*** At early time-points invasive growth was primarily evident as a darkening of single-cell derived colonies that had not yet fused together in the '5x' dilution spot. (These phenotypes were sometimes more evident from direction inspection of the plates than they were in the photographs.) At later stages invasion was only evident when peripheral to the main colony. While evidence for invasion was scored as 'enhanced' morphology, we are more confident in the direct assays of invasive growth used in the primary screen and subsequent follow-up analysis.
- ***Inter-batch variation in morphology development:*** Colony morphology development is influenced by subtle variation in a variety of factors, including pH, nutrient concentrations, temperature, and even the moistness of the plate surface. Thus, it was not surprising that we encountered some variation in the extent of colony morphology formation among batches of plates. Since our scores are assigned relative to plate-specific wild-type controls, this variation did not lead to false positives per se, but did result in the debatable attribution of the '[I]' flag (see above) to some morphology scores. This problem was particularly evident with our YEPD plates. We suspect subtle variation pH as the underlying cause of the variation. The pH of these plates was ~6.3, which lies on the cusp of the neutral pH that is often used to promote *C. albicans* filamentation.

4. Detection limits

Because all scoring is relative to wild-type, the growth and morphological state of the wild-type strain on a given condition dictates whether enhancement or reduction of phenotype is detectable. For example, a stress condition that nearly abolishes wild-type growth facilitates the detection of growth enhancement of the mutant strain but renders growth reduction undetectable. Thus, when data are provided for two different concentrations of a stress agent, it may at first seem anomalous that a growth defect is observed for the low concentration but not the high concentration. We refer the reader to the phenotype viewing software to view the wild-type growth and morphological state for each condition.

4. Arginine auxotrophy

Our strain background lacks a functional *ARG4* gene. Thus, in the case of some screens of nutrient utilization, it is possible that the lack of a functional arginine biosynthetic pathway may either contribute to or mask phenotypes. We note that the lack of *ARG4* did not affect the phenotypes observed for our *ΔΔorf19.4766* TRKO, even though *ORF19.4766* is the ortholog of the *S. cerevisiae* arginine circuit

regulator *ARG81*. Reintroduction of a single *ARG4* allele into the knockout strain eliminated the arginine auxotrophy but did not alter the observed phenotypes (data not shown).

5. Inter-isolate variability

Given that the process of creating a deletion strain can be mutagenic, it is not surprising that independent isolates of a particular mutant sometimes exhibited reproducible but inconsistent phenotypes. When possible, one or more additional isolates were created to reconcile the inconsistencies, with the assumption that the isolates exhibiting consistent phenotypic profiles reflect the true phenotypic consequences of the mutation. However, in some cases additional isolates were not available or the additional isolates did not reconcile the phenotypic differences. Thirteen mutants were excluded from the analysis for this reason (see Data Set S2), leading to our rough estimate that up to 10% of the library isolates contained secondary mutations that yielded phenotypes not associated with the TR knockout. We note that more subtle inter-isolate variation was sometimes seen even among mutants that were included in the final analysis. However, without many additional phenotyping replicates we cannot determine whether this variability is due inter-isolate genetic variation or some of the additional assay limitations highlighted above.

Supplemental Phenotyping Protocols

While the methodology for the final phenotyping screen is provided in the primary Materials and Methods, here we provide details on the protocols utilized for the supplemental screens (and elaborate on the invasive growth screen utilized in the primary screen).

1. Invasive Growth (primary screen and supplemental)

The invasive growth assay in the primary screen involved the additional step of washing the plate surface with water to determine whether cells were embedded in the agar. YEPD plates were washed after two days of growth at 30°C and SD plates were washed after three days of growth at 30°C. After the wash step, the plates were photographed, incubated for an additional day at 30°C, and photographed once more. The plates were often overgrown after this continued incubation, but this final image was sometimes useful in detecting reduced invasion phenotypes. The invasion score was based on the photograph taken immediately after washing.

The approach taken in the primary screen provided a snapshot of invasion at only one time-point and temperature. A more extensive and rigorous supplemental screen was conducted to provide more a more detailed description of invasion phenotypes. All TRKO strains that exhibited moderate or stronger invasive growth phenotypes in the primary screen were retested (with the exception of the hyper-invasive *ΔΔnrg1* and *ΔΔtup1* mutants). Strains were grown and plated as described for the primary screen, with the following exceptions:

1. In order to minimize proximity effects, the strains were plated only as '1x' dilutions in a checkerboard pattern such that each macro-colony was only orthogonal to other macro-colonies.
2. In order to control for edge effects and determine consistency, several wild-type macro-colonies were included on each plate at both central and perimeter locations.
3. The assay was conducted at both 30˚C and 37˚C.
4. Multiple plates were 'stamped' with the pin tool such that plates could be washed at multiple time-points: 2, 3, and 6 days. The invasion scores provided for these assays reflect the strongest score across all tested time-points.
5. Other media were tested, including Spider and YEPD buffered with HEPES to pH7.3 (to reduce any contribution of ammonia pulses to invasion and proximity effects). The Spider media did not promote invasion, and the buffered YEPD yielded results indistinguishable from the unbuffered YEPD plates.

6. The post-washing incubation step was omitted, as it was not particularly informative.

2. Single-Cell-Colony Morphologies

Because the colony morphologies observed in the primary screen were not derived from single cells, subtle differences in colony morphology were difficult to describe. In order to further characterize knockout strains that exhibited colony morphology phenotypes on Spider, we plated each strain for single-cell-derived colonies on YEPD, Spider at (30°C and 37°C) and CHROMagar media (37°C). The strains were prepared for plating as described for the primary screen, with the exception that the final dilution was calibrated to yield 5-30 colonies when spread onto a plate using glass beads. A representative colony from each plate was photographed after three and seven days of growth (not necessarily the same colony). On the rare occasions when variable colony morphologies were observed on a plate, the less abundant form was included as an inset to the photo of the predominant colony morphology. Images were taken using a Nikon CoolPix 4300 camera affixed to a Nikon SMZ-U stereoscopic microscope. As all photos for a given day were taken at approximately the same magnification level, colony size differences evident in the phenotype viewing software are likely to reflect differences in growth rate.

We experienced some inconsistency in colony morphology phenotypes with our wild-type replicates on YEPD (possibly influenced by plate moisture levels), and the CHROMagar morphologies were complex and difficult to characterize. Therefore, although we include these data in the viewing software, we focused on the more robust Spider medium for further analysis. While these phenotypes were scored in the viewing software so that they could be searched by the end-user, we also scored these phenotypes outside of the software using a scheme that separately considered peripheral filamentation and the structure of the primary colony (Data Set S2). We note that even in the case of the more reliable Spider medium, some variation in the colony morphology at 30°C on day 7 was observed between batches of plates. Some plate batches yielded full colony wrinkling of wild-type strains on this condition, while others did not. The colony morphology shown for the wild-type strain in Figure 3 reflects the latter variant. All TRKO strains shown as exhibiting enhanced wrinkling on this condition were tested on the same plate batch as the wild-type strain and exhibited the phenotype for both isolates.

3. Straight-from-Stock (low sensitivity assays)

The strains in the TRKO collection were stored as glycerol stocks in a 96-well format. In a few cases (CHROMagar medium, anaerobic growth, Isoleucine as nitrogen source), phenotypes were assayed by directly transferring cells from the glycerol stock to the test medium using a 48-pin bolt replicator. This approach was considered 'low-sensitivity' because the transfer directly from stock precluded the careful control over the number of cells deposited. Under these conditions, strong phenotypes were reproducible between TRKO isolates, but weaker phenotypes may have escaped detection.

4. Targeted Supplemental Phenotyping

In some cases, a TRKO was assayed for the presence of a phenotype identified for an orthologous gene (e.g. capacity of *ΔΔorf19.4766*, the ortholog of *S. cerevisiae ARG81*, to utilize ornithine as a nitrogen source). In such cases, the methodology utilized was either a variation of the dilution series approach utilized in the primary screen (often with addition dilutions) or the more direct approach of streaking the strains on a plate (i.e. "wedge-shaped" deposition of cells).

5. S. cerevisiae TRKO phenotyping screen

The methodology utilized for phenotyping of *S. cerevisiae* TRKO strains was identical to that of the primary *C. albicans* screen.

Phenotype Validation

The overall quality of the phenotypic data obtained from the primary screen was assessed using several approaches:

1. Reproducibility Of Additional Replicates

The primary screen assayed each combination of TRKO and growth condition at least two times (once per isolate). The supplemental screens provided a further test of reproducibility, and included repeat measurements of caffeine, 5-Fluorocytosine, copper, EDTA, and fluconazole phenotypes for TRKOs that exhibited phenotypes in the primary screen on these media. As shown in Data Set S2, these follow-up assays revealed near perfect reproducibility of the previously observed phenotypes (though the precise intensity of the phenotypes varied to some degree). In the case of copper medium, we note that the concentration of copper in the stock solution utilized in the follow-up assay appeared to differ from that used in the original assay. This initially led to poor reproducibility of the previously observed phenotypes, but upon testing of a range of copper concentrations, the copper phenotypes of the primary screen were recapitulated.

2. Overlapping Phenotypic Profiles of TRKOs Associated With Protein Complexes

ORF19.2088 and *ORF19.3063* both resemble *S. cerevisiae* genes annotated in the Saccharomyces Genome Database[2] as subunits of the DNA Polymerase B (II) subunit. The two deletion mutants exhibited virtually identical phenotypic profiles, a result consistent with disruption of a protein complex and suggesting a low incidence of incorrect phenotype assignment. Similarly, deletion mutants of *HAP2* and *HAP5*, both believed to be members of the CCAAT-binding complex, yielded nearly identical phenotypic profiles.

3. Reproducibility of Previously Reported Phenotypes

While it is beyond the scope of this study to fully document all *C. albicans* TRKO phenotypes reported in existing literature, whenever possible we noted previous reports of phenotypes in the TRKO phenotype summaries (see Data Set S2). In nearly every case the reported phenotype was recapitulated in our assay.

Orthology: Classification of Orthologs

Orthologs of *C. albicans* and *S. cerevisiae* genes were identified by comparative analysis of ortholog mappings obtained from two sources:

1. **SYNERGY**[3] algorithm: courtesy of The Fungal Orthogroups Repository (http://www.broad.mit.edu/regev/orthogroups/)
2. **InParanoid**[4] algorithm using BLOSUM80 and an InParanoid score of 100%: courtesy of the Candida Genome Database[5] (www.candidagenome.org)

The CGD mapping also included "**Best Hit**" calls for genes that were not assigned an ortholog using InParanoid. These calls were generated by BLASTp with a minimum expectation value of 1e-05.

A script was written to compare the **SYNERGY** and **'InParanoid+Best Hit'** mappings. Because of the very strict settings utilized by CGD when running **InParanoid**, orthology relationships more complex than 1-to-1 were rarely identified. Thus, emphasis was given to the **SYNERGY** results and **InParanoid** results were used as additional confirmation. The comparison script also utilized the list of *S. cerevisiae* gene pairs ("ohnologs") derived from the Whole Genome Duplication (WGD) event compiled by Byrne et al.[6]. These data were used to specifically flag 1-to-2 mappings that reflect this evolutionary duplication event. Below, we summarize the possible outcomes of this comparison and the format utilized to summarize the information in the supplemental data.

Note: In all cases where orthologous relationships are described as #-to-#, the first number refers to C. albicans and the second to S. cerevisiae.

No ortholog or best hit from either dataset (N/A)

Genes falling into this category were interpreted as having no ortholog in the other species.

1-to-1 ortholog mapping in both SYNERGY and 'InParanoid+Best Hit' (Clear_1to1)

These cases were interpreted as clear evidence of orthology. Note that this criterion includes cases with a 1-to-1 **best hit** mapping (i.e. The strict settings used in **InParanoid** failed to identify the ortholog, but the more relaxed BLASTp search supported the **SYNERGY** orthology determination).

1-to-2 orthology supported by Whole Genome Duplication data (Clear_1to2_WGD)

Instances of 1-to-2 orthology that reflects the *S. cerevisiae* WGD event were considered to be clear-cut when the 1-to-2 relationship was properly identified by **SYNERGY** and ***'InParanoid+Best Hit'*** exclusively identified at least one of the two *S. cerevisiae* genes as the *C. albicans* ortholog or **best hit**.

More complex or ambiguous results

These results were represented by a set of concatenated summary data:

1. *[SYN:#-to-#]* or *[SYN:None]*: the orthology mapping from **SYNERGY**
2. *[IPo:#-to-#]* or *[IPbh:#-to-#]* or *[IP:None]*: the orthology mapping from **InParanoid** (IPo) or the **best hit** (IPbh)
3. *[Ca:Same]* or *[Ca:Overlap]* or *[Ca:Subset]* or *[Ca:Different]*: In cases where both **SYNERGY** and **InParanoid** yielded ortholog calls, this tag summarizes the intersection of the *C. albicans* genes called by the two approaches
4. *[Sc:Same]* or *[Sc:Overlap]* or *[Sc:Subset]* or *[Sc:Different]*: As above, but for *S. cerevisiae* genes.

Hand-annotated complex or ambiguous results

In some instances, complex or ambiguous orthology relationships were further analyzed by comparison to gene trees generated from alignments of similar genes from 32 fungal genomes as identified by PSI-BLAST of each *C. albicans* gene and each *S. cerevisiae* gene (courtesy of Brian Tuch; data not shown). In these cases the inferred relationship was annotated by hand and superseded the results generated by the other datasets.

Orthology: Comparative Phenotype Analysis

In order to compare the phenotypes of our *C. albicans* TRKOs to those of *S. cerevisiae* orthologs, we compiled a detailed listing of phenotypes attributed to *S. cerevisiae* genes using data from the following sources:

1. **Saccharomyces Genome Database**[2]: Data were extracted from the phenotype file available at ftp://ftp.yeastgenome.org/yeast/literature_curation/phenotypes.tab. Note that this file predates the July reorganization of the SGD phenotype nomenclature.
2. **Chemical Genomic Portrait**[7]: The extensive phenotyping data from this liquid medium study were condensed to provide a single value for each condition tested. This was deemed necessary to identify robust phenotypes. For example, the homozygous deletion library was tested with rapamycin 7 times, using a variety of generation times and drug concentrations. Robust phenotypes were identifying by taking the median of the p-values for each gene (and requiring that at least half of the conditions had a non-blank entry).
3. **Solid Media Phenotypes**[8]: This study included analyses of caffeine, rapamycin, and BPS.
4. **Zinc Cluster Protein Phenotyping**[9]: This study included a meta-analysis of existing phenotyping data.
5. **TOR Phenotypes On Cell Array**[10]: These data included rapamycin phenotyping.
6. **Heterozygote Phenotyping**[11]: A liquid medium phenotyping assay that included the following conditions: caffeine, menadione, fenpropimorph, and 5-fluorocytosine.
7. **Metal Phenotypes**[12]: This study assayed copper and iron sensitivity in liquid media.

Ultimately, we determined that the highly diverse methodologies (and drug concentrations) of these assays limited their utility in cross-species comparison. For example, comparison of rapamycin sensitivity phenotypes among the studies revealed poor correspondence between *S. cerevisiae* assays. Thus, while these data were used to guide our initial investigation of inter-species phenotypic conservation, we also conducted our own phenotyping assays utilizing the same methodology used for our primary *C. albicans* phenotyping screen.

References

1. Eisen MB, Spellman PT, Brown PO, Botstein D (1998) Cluster analysis and display of genome-wide expression patterns. Proc Natl Acad Sci U S A 95: 14863-14868.
2. SGD Saccharomyces Genome Database.
3. Wapinski I, Pfeffer A, Friedman N, Regev A (2007) Automatic genome-wide reconstruction of phylogenetic gene trees. Bioinformatics 23: i549-558.
4. Remm M, Storm CE, Sonnhammer EL (2001) Automatic clustering of orthologs and in-paralogs from pairwise species comparisons. J Mol Biol 314: 1041-1052.
5. Costanzo MC, Arnaud MB, Skrzypek MS, Binkley G, Lane C, et al. (2006) The Candida Genome Database: facilitating research on Candida albicans molecular biology. FEMS Yeast Res 6: 671-684.
6. Byrne KP, Wolfe KH (2005) The Yeast Gene Order Browser: combining curated homology and syntenic context reveals gene fate in polyploid species. Genome Res 15: 1456-1461.
7. Hillenmeyer ME, Fung E, Wildenhain J, Pierce SE, Hoon S, et al. (2008) The chemical genomic portrait of yeast: uncovering a phenotype for all genes. Science 320: 362-365.
8. Dudley AM, Janse DM, Tanay A, Shamir R, Church GM (2005) A global view of pleiotropy and phenotypically derived gene function in yeast. Mol Syst Biol 1: 2005 0001.
9. Akache B, Wu K, Turcotte B (2001) Phenotypic analysis of genes encoding yeast zinc cluster proteins. Nucleic Acids Res 29: 2181-2190.
10. Xie MW, Jin F, Hwang H, Hwang S, Anand V, et al. (2005) Insights into TOR function and rapamycin response: chemical genomic profiling by using a high-density cell array method. Proc Natl Acad Sci U S A 102: 7215-7220.
11. Lum PY, Armour CD, Stepaniants SB, Cavet G, Wolf MK, et al. (2004) Discovering modes of action for therapeutic compounds using a genome-wide screen of yeast heterozygotes. Cell 116: 121-137.
12. Jo WJ, Loguinov A, Chang M, Wintz H, Nislow C, et al. (2008) Identification of genes involved in the toxic response of Saccharomyces cerevisiae against iron and copper overload by parallel analysis of deletion mutants. Toxicol Sci 101: 140-151.
