# Supplementary material for: A Phenotypic Profile of the Candida albicans Regulatory Network
Source: PLoS Genet. 2009 Dec 24;5(12):e1000783. doi: 10.1371/journal.pgen.1000783 (PMC2790342; doi:10.1371/journal.pgen.1000783)
Supplement: Text S3 — Analysis of TOR pathway phenotypes. (0.35 MB PDF) [file pgen.1000783.s003.pdf]

An overview of the role of TOR pathway in nutrient signaling is provided in the primary manuscript. Here, we describe many of the individual transcriptional regulators (TRs) that exhibited strong caffeine sensitivity and resistance phenotypes. The majority of these TRs can be connected to nutrient signaling by comparison to *S. cerevisiae* orthologs (or homologs). Moreover, a subset of these TRs appear to constitute a conserved core set of regulators[1] that directly control TOR function in both *S. cerevisiae* and *C. albicans* (Table 1, below).

Eight *C. albicans* TRKO strains exhibited enhanced resistance to caffeine (Figure 2B). Seven of the eight regulators have known roles in nutrient signaling: *GAT1* and *GLN3* are key TOR-responsive regulators of nitrogen catabolite repression in both *S. cerevisiae*[2] and *C. albicans*[3-5]. *STP3* is a regulator of peptide uptake in *C. albicans* [6,7], and *DAL81 (ORF19.3252)* is an uncharacterized *C. albicans* ortholog of the *S. cerevisiae DAL81* gene, which promotes nitrogen catabolism in *S. cerevisiae*[8] (*ΔΔdal81* is not included in Figure 2B because the knockout isolates were not fully independent). Three more caffeine-resistant TRKOs, *HAP5*, *ORF19.1228*, and *HAP31*, are thought to be members of the CCAAT-binding complex. This complex regulates respiration and carbon metabolism in both *S. cerevisiae[9]* and *C. albicans[10,11]*. We assayed deletion mutants of the three *S. cerevisiae* orthologs *HAP2*, *HAP3*, and *HAP5*, and found that all were resistant to rapamycin (Data Set S3).

The final caffeine-resistant TRKO, *ΔΔorf19.4166*, was atypical of the other caffeine-resistant strains in two key respects. First, *ORF19.4166* was the only caffeine-resistant TR that lacked significant similarity to a *S. cerevisiae* TR. This TR has not been studied in *C. albicans*, and has no clear link to nutrient metabolism. Second, *ΔΔorf19.4166* was the only TRKO strain that exhibited resistance to caffeine but not rapamycin (several rapamycin concentrations were tested). Based on these observations, we suggest that *ORF19.4166* may regulate processes that specifically influence the import, export, or degradation of caffeine.

We identified 14 caffeine-sensitive TRKOs. Five of these - *ΔΔtup1*, *ΔΔgzf3*, *ΔΔrim101*, *ΔΔndt80*, and *ΔΔbcr1* – also exhibited strong morphology phenotypes and sensitivity to 0.3M LiCl. Several lines of evidence suggest a mechanistic connection between these phenotypes. In *S. cerevisiae*, the TOR pathway has been shown to influence cell wall integrity, membrane trafficking, and actin polarization – all pathways that are likely to be critical for morphogenesis. In *C. albicans*, Tor1 has also been implicated in the regulation of hyphal genes such as adhesins[12]. (This study also demonstrated rapamycin-sensitivity of *ΔΔtup1* and *ΔΔnrg1* mutants, phenotypes also observed in our study.) In addition, in both *C. albicans* and *S. cerevisiae* rapamycin has been shown to inhibit filamentous growth on nitrogen-poor media[13]. In *C. albicans*, lithium also inhibits filamentation (on galactose-containing media)[14]. Furthermore, the calcineurin pathway, known to interact with TOR in *S. cerevisiae*[15], influences lithium-ion tolerance in *S. cerevisiae[16]* and both lithium tolerance and colony morphology in *C. albicans*[17]. Thus, the existing literature documents connections between TOR function and both morphogenesis and lithium ions, and also documents a connection between lithium ions and morphogenesis. Our phenotypic data reinforce these connections, and underscore the influence of the five TRs listed above on multiple regulatory circuits in the cell.

The remaining nine caffeine-sensitive TRKOs exhibited a variety of phenotypic profiles, and included three TRs with likely or confirmed roles in nutrient response: *ΔΔmig1*, *ΔΔorf19.2961* , and *ΔΔorf19.4766*. (For completeness, we note that the *S. cerevisiae* ortholog of *GZF3*, mentioned in the previous paragraph, also has a known role in nutritional/TOR signaling[1].) *MIG1* is a clear ortholog of the *S. cerevisiae* genes *MIG1* and *MIG2*; in the presence of glucose, both *S. cerevisiae MIG* genes repress the expression of genes involved in the utilization of non-preferred carbon sources[18]. *ORF19.2961* is

previously uncharacterized, but is similar to *S. cerevisiae MIG1/MIG2.* Based on the phenotype of the *ΔΔorf19.2961* mutant, we propose that Orf19.2961 plays a regulatory role similar to Mig1/Mig2. The third caffeine-sensitive TRKO with a connection to nutrient response was *ΔΔorf19.4766*. This TR is the ortholog of *S. cerevisiae ARG81*, a repressor of the arginine biosynthesis genes. The TRKO shares two traits that confirm that it is the ortholog of *S. cerevisiae ARG81*: (1) it is unable to utilize ornithine as a nitrogen source (Data Set S2) and (2) it shows strong up-regulation of arginine biosynthetic genes (WT vs. TRKO expression data from cultures grown in YEPD at 30°C; data not shown). However, the *S. cerevisiae ARG81* mutant exhibits only very weak sensitivity to caffeine (Data Set S3), suggesting that the transcriptional network of *C. albicans* exhibits a stronger connection between the arginine regulatory circuit and the TOR pathway.

Finally, we note that two caffeine-sensitive TRKOs, *ΔΔorf19.1168* and *ΔΔorf19.5133*, have no clear *S. cerevisiae* counterpart, and may represent regulators of the TOR pathway that are absent in that species.

Table 1. The six transcriptional regulators identified by Bertram et al.[1] as physically interacting with Tor1 either have an ortholog in *C. albicans* with a phenotype reflecting a role in TOR function or lack an ortholog entirely. All reported phenotypes are from this study and are described in Data Sets S2 and S3.

S. cerevisiae Gene	*C. albicans* Gene	*C. albicans* Caffeine Phenotype	*C. albicans* Rapamycin Phenotype	*S. cerevisiae* Caffeine Phenotype	*S. cerevisiae* Rapamycin Phenotype	**Comments**
GZF3 / DAL80	*GZF3*	Sensitive	Sensitive	N/A	N/A	In S. cerevisiae, *GZF3* and *DAL80* are products of the whole genome duplication.
GAT1	*GAT1*	Resistant	Resistant	Resistant	Resistant	
GLN3	*GLN3*	Resistant	Resistant	Resistant	Resistant	
DAL81	*orf19.3252*	Resistant	Resistant	Resistant	N/A	Not included in Figure 2 of the manuscript because only a single mutant isolate was created.
DAL82		N/A	N/A	N/A	N/A	*DAL82* lacks a clear ortholog in *C. albicans*

References

1. Bertram PG, Choi JH, Carvalho J, Ai W, Zeng C, et al. (2000) Tripartite regulation of Gln3p by TOR, Ure2p, and phosphatases. J Biol Chem 275: 35727-35733.
2. Beck T, Hall MN (1999) The TOR signalling pathway controls nuclear localization of nutrient-regulated transcription factors. Nature 402: 689-692.
3. Dabas N, Morschhauser J (2007) Control of ammonium permease expression and filamentous growth by the GATA transcription factors GLN3 and GAT1 in Candida albicans. Eukaryot Cell 6: 875-888.
4. Liao WL, Ramon AM, Fonzi WA (2008) GLN3 encodes a global regulator of nitrogen metabolism and virulence of C. albicans. Fungal Genet Biol 45: 514-526.
5. Limjindaporn T, Khalaf RA, Fonzi WA (2003) Nitrogen metabolism and virulence of Candida albicans require the GATA-type transcriptional activator encoded by GAT1. Mol Microbiol 50: 993-1004.
6. Martinez P, Ljungdahl PO (2005) Divergence of Stp1 and Stp2 transcription factors in Candida albicans places virulence factors required for proper nutrient acquisition under amino acid control. Mol Cell Biol 25: 9435-9446.
7. Dabas N, Morschhauser J (2008) A transcription factor regulatory cascade controls secreted aspartic protease expression in Candida albicans. Mol Microbiol 69: 586-602.
8. Bricmont PA, Daugherty JR, Cooper TG (1991) The DAL81 gene product is required for induced expression of two differently regulated nitrogen catabolic genes in Saccharomyces cerevisiae. Mol Cell Biol 11: 1161-1166.
9. Pinkham JL, Guarente L (1985) Cloning and molecular analysis of the HAP2 locus: a global regulator of respiratory genes in Saccharomyces cerevisiae. Mol Cell Biol 5: 3410-3416.
10. Johnson DC, Cano KE, Kroger EC, McNabb DS (2005) Novel regulatory function for the CCAAT-binding factor in Candida albicans. Eukaryot Cell 4: 1662-1676.
11. Baek YU, Li M, Davis DA (2008) Candida albicans ferric reductases are differentially regulated in response to distinct forms of iron limitation by the Rim101 and CBF transcription factors. Eukaryot Cell 7: 1168-1179.
12. Bastidas RJ, Heitman J, Cardenas ME (2009) The protein kinase Tor1 regulates adhesin gene expression in Candida albicans. PLoS Pathog 5: e1000294.
13. Cutler NS, Pan X, Heitman J, Cardenas ME (2001) The TOR signal transduction cascade controls cellular differentiation in response to nutrients. Mol Biol Cell 12: 4103-4113.
14. Martins LF, Montero-Lomeli M, Masuda CA, Fortes FS, Previato JO, et al. (2008) Lithium-mediated suppression of morphogenesis and growth in Candida albicans. FEMS Yeast Res 8: 615-621.
15. Mulet JM, Martin DE, Loewith R, Hall MN (2006) Mutual antagonism of target of rapamycin and calcineurin signaling. J Biol Chem 281: 33000-33007.
16. Mendoza I, Rubio F, Rodriguez-Navarro A, Pardo JM (1994) The protein phosphatase calcineurin is essential for NaCl tolerance of Saccharomyces cerevisiae. J Biol Chem 269: 8792-8796.
17. Sanglard D, Ischer F, Marchetti O, Entenza J, Bille J (2003) Calcineurin A of Candida albicans: involvement in antifungal tolerance, cell morphogenesis and virulence. Mol Microbiol 48: 959-976.
18. Santangelo GM (2006) Glucose signaling in Saccharomyces cerevisiae. Microbiol Mol Biol Rev 70: 253-282.
