# Supplementary material for: A Phenotypic Profile of the Candida albicans Regulatory Network
Source: PLoS Genet. 2009 Dec 24;5(12):e1000783. doi: 10.1371/journal.pgen.1000783 (PMC2790342; doi:10.1371/journal.pgen.1000783)
Supplement: Text S4 — Analysis of copper/iron homeostasis phenotypes. (0.29 MB PDF) [file pgen.1000783.s004.pdf]

TEXT S4. Analysis of copper/iron homeostasis phenotypes

A phenotypic description of the regulatory network governing copper and iron homeostasis is provided in the primary manuscript. Here we discuss our findings in greater detail, with an emphasis on aspects relevant to the evolution of the regulatory circuit in fungi.

There are several important differences between the *C. albicans* and *S. cerevisiae* iron regulation circuitry, the most prominent being that the iron uptake circuit is positively regulated by Aft1 in *S. cerevisiae* and negatively regulated by Sfu1 in *C. albicans*. *S. cerevisiae* lacks an *SFU1* ortholog. *C. albicans* contains a possible ortholog of *S. cerevisiae AFT1*, *ORF19.2272*, but we found that deletion of this ORF does not result in the BPS-sensitivity characteristic of the *ΔΔaft1 S. cerevisiae* strain (Data Set S3, and data not shown). *S. cerevisiae* has a paralog of *AFT1*, called *AFT2*, a product of the *Saccharomyces* branch whole genome duplication event. The *ΔΔaft2 S. cerevisiae* strain is not BPS-sensitive and is thought to be an intra-cellular regulator of iron homeostasis[1,2]. Given that neither the *C. albicans ΔΔof19.2272* nor the *S. cerevisiae ΔΔaft2* knockouts showed BPS-sensitivity, it is possible that *AFT2* retains the ancestral function of the AFT gene that preceded the *AFT1/AFT2* duplication event.

Our screen also uncovered a defect in iron acquisition in a *C. albicans* TR that underwent a duplication event in the *Candida* branch of the ascomycetes. Deletion of the *C. albicans* TR *SEF1*, but not the paralog *SEF2*, resulted in BPS-sensitivity. The *SEF2* paralog may also regulate metal homeostasis: deletion of this gene resulted in copper sensitivity. Although the *SEF1/SEF2* ortholog in *S. cerevisiae* (called *SEF1*) had no previously reported role in iron homeostasis, we find that a *S. cerevisiae ΔΔsef1* strain is also BPS-sensitive. Thus, the BPS-sensitivity phenotype of *C. albicans SEF1* may reflect the ancestral function of the *SEF* genes. The role of *SEF1* appears to be conserved between *S. cerevisiae* and *C. albicans*, and we note the intriguing possibility that the duplication event that yielded *SEF2* in the *C. albicans* lineage allowed further refinement of the circuit in a manner reminiscent of the *AFT1/AFT2* duplication in *S. cerevisiae*.

In *S. cerevisiae*, *SEF1* exhibits a genetic interaction with the CCAAT-complex genes *HAP3, HAP4*, and *HAP5* (Nevan Krogan, personal communication). Interestingly, we (and others[3,4]) noted BPS-sensitivity in *C. albicans* CCAAT-complex members (*HAP43*, *ORF19.1228*, *HAP5*), but not *S. cerevisiae* complex members (*HAP2, HAP3,* and *HAP5* were tested; Data Set S3). Thus, while the *S. cerevisiae* CCAAT-complex is indirectly linked to iron response via the scΔ*sef1* phenotype, the phenotypic consequence of CCAAT-complex deletion appears to have diverged in the two species. These differences could reflect altered regulation of respiration, a process that increases demand for iron[5], by the CCAAT-complex in the two species. Indeed, there is some evidence that the circuit is wired differently in the two species [3,4], but a definitive comparison utilizing chromatin immunoprecipitation has yet to be conducted.

References

1. Blaiseau PL, Lesuisse E, Camadro JM (2001) Aft2p, a novel iron-regulated transcription activator that modulates, with Aft1p, intracellular iron use and resistance to oxidative stress in yeast. J Biol Chem 276: 34221-34226.
2. Courel M, Lallet S, Camadro JM, Blaiseau PL (2005) Direct activation of genes involved in intracellular iron use by the yeast iron-responsive transcription factor Aft2 without its paralog Aft1. Mol Cell Biol 25: 6760-6771.
3. Johnson DC, Cano KE, Kroger EC, McNabb DS (2005) Novel regulatory function for the CCAAT-binding factor in Candida albicans. Eukaryot Cell 4: 1662-1676.
4. Baek YU, Li M, Davis DA (2008) Candida albicans ferric reductases are differentially regulated in response to distinct forms of iron limitation by the Rim101 and CBF transcription factors. Eukaryot Cell 7: 1168-1179.
5. Philpott CC, Protchenko O (2008) Response to iron deprivation in Saccharomyces cerevisiae. Eukaryot Cell 7: 20-27.
6. Ogasawara A, Odahara K, Toume M, Watanabe T, Mikami T, et al. (2006) Change in the respiration system of Candida albicans in the lag and log growth phase. Biol Pharm Bull 29: 448-450.
7. Schuller HJ (2003) Transcriptional control of nonfermentative metabolism in the yeast Saccharomyces cerevisiae. Curr Genet 43: 139-160.
