# Supplementary material for: A Phenotypic Profile of the Candida albicans Regulatory Network
Source: PLoS Genet. 2009 Dec 24;5(12):e1000783. doi: 10.1371/journal.pgen.1000783 (PMC2790342; doi:10.1371/journal.pgen.1000783)
Supplement: Text S5 — Phenotypic comparison between C. albicans and S. cerevisiae orthologs. (0.45 MB PDF) [file pgen.1000783.s005.pdf]

# TEXT S5. Phenotypic comparison between *C. albicans* and *S. cerevisiae* orthologs

The primary manuscript provides a general outline of phenotypic conservation between *Saccharomyces cerevisiae* and *Candida albicans* transcriptional regulator orthologs. Below, we provide a listing of the specific phenotypes being compared, including an indication of the phenotype source (i.e. Data Sets S2 and S3 from this study as well as references from the existing literature). The data are split into two tables. Table 1 describes orthologs with a 1-to-1 relationship, and Table 2 describes orthologs with a 1-to-2 relationship that arose from the *S. cerevisiae* whole genome duplication.

**Table 1.** Phenotype conservation between S. cerevisiae and C. albicans transcriptional regulators with 1-to-1 orthology.

| *S. cerevisiae* Gene | *C. albicans* Gene | Shared Phenotypes | Dissimilar Phenotypes | Comments |
|---|---|---|---|---|
| *Dissimilar Orthologs* | | | | |
| *GAL4*† | *GAL4* | | *Sc:* Galactose as C-source[1,2]* | |
| *ISW2* | *ISW2* | | *Ca:* Fluconazole sensitivity§* | |
| *MBP1* | *ORF19.5855* | | *Sc:* Base media growth impairment; Caffeine and Rapamycin resistant [3]§* | |
| *STB5* | *ORF19.3308* | | *Sc:* Base media growth impairment and Caffeine sensitivity[4,5]§* | |
| *RTG1*† | *ORF19.4722* | | *Sc:* Glutamate and Aspartate auxotrophy and Acetate as C-source[6], base SC medium growth impairment (lacks Glutamate and Aspartate)§ | Orthology may be more complex than 1-to-1. |
| *Mixed Similarity Orthologs* | | | | |
| *DPB4* | *ORF19.2088* | Base media growth impairment in liquid[3]* medium | *Ca:* Base media growth impairment on solid medium§* | |
| *ARG81* | *ORF19.4766* | Ornithine as N-source[7]* | *Ca:* Strong caffeine sensitivity§*; *Sc:* Very weak caffeine sensitivity | |
| *BAS1* | *BAS1* | Adenine auxotrophy[8]* | *Sc:* YEPD growth impairment[3]§* | |
| *GLN3*¥ | *GLN3* | Caffeine and Rapamycin resistance and Base SD medium growth impairment[9]§* | *Sc:* Base YEPD medium growth impairment[3]§* | |
| *HAP2*† | *ORF19.1228* | Non-glucose carbon source utilization[10]* | *Ca:* BPS sensitivity§* | |
| *TUP1* | *TUP1* | Flocculation[11,12]* | *Ca:* Fluconazole and Caffeine sensitivity; *Sc:* YEPD growth impairment[3]§* | Extensively characterized in both species and highly pleiotropic. |
| *ZAP1*† | *CSR1* | EDTA sensitivity[13]§* | *Sc:* Base media growth impairment[3]§* | |
| *HAP5*† | *HAP5* | Non-glucose carbon source utilization[10]* | *Ca:* BPS sensitivity§* | |
| *Similar Orthologs* | | | | |
| *ARO80*Ω | *ARO80* | Isoleucine as N-source[14]* | | *S. cerevisiae* phenotype is inferred based on expression profiling data. |
| *CYC8* | *SSN6* | Base media growth impairment[3]§*, etc. | | Extensively characterized in both species and highly pleiotropic. |

| *S. cerevisiae* Gene | *C. albicans* Gene | Shared Phenotypes | Dissimilar Phenotypes | Comments |
|---|---|---|---|---|
| *DAL81*[Ω] | *ORF19.3252* | Base media growth impairment[5]§* | | |
| *GAT1*[¥] | *GAT1* | Caffeine and Rapamycin resistance[9,15] §* | | |
| *HCM1* | *HCM1* | Base media growth impairment[3]§* | | Growth impairment is more severe in *C. albicans* mutant. |
| *MAC1* | *MAC1* | Metal homeostasis[16,17]§* | | |
| *PHO2*[†] | *ORF19.4000* | Leaky adenine auxotrophy[18] §* | | |
| *PHO4*[†] | *ORF19.1253* | Low phosphate sensitivity*§ | | |
| *RDS2* | *CWT1* | Calcofluor White sensitivity[4,19] | | |
| *RGT1*[†] | *RGT1* | Glucose transport [20,21] | | Function primarily inferred from regulatory data, not from phenotype. |
| *RIM101*[¥] | *RIM101* | Sensitivity to alkaline pH[22,23]* | | Highly pleiotropic. Too many phenotypes to fully catalog. |
| *UPC2*[‡] | *UPC2* | Impaired anaerobic growth and sensitivity to Fluconazole[24-26]§* | | Orthology may be more complex than 1-to-1 (not included in manuscript table). |
| *UGA3*[‡] | *ORF19.7570* | GABA as N-source[27]* | | Orthology appears to be more complex than 1-to-1 (not included in manuscript table). |
| *SFL1*[‡] | *SFL1* | Invasive growth, flocculation, metal homeostasis[28-31]§* | | Orthology appears to be more complex than 1-to-1 (not included in manuscript table). |

[†] These genes are classified as orthologs using SYNERGY but are classified as "Best Hits" in CGD.
[‡] These genes are classified as orthologs in CGD but lacked a clear ortholog using SYNERGY.
[¥] Hand-annotated orthology
*C. albicans* phenotype assayed in this study
§ *S. cerevisiae* phenotype assayed in this study
[Ω] *C. albicans* knockout isolates were not fully independent.

**Table 2.** Phenotype conservation between S. cerevisiae and C. albicans transcriptional regulators with 1-to-2 orthology.

| *S. cerevisiae* WGD Genes | *C. albicans* Gene | Shared Phenotypes | Dissimilar Phenotypes | Comments |
|---|---|---|---|---|
| *Dissimilar Orthologs* | | | | |
| *YML081W + RSF2* | *ORF19.5026* | | *scYML081W*: Impaired growth on base media*§ | The mutant phenotype of *scYML081W* was not reported in other high-throughput studies. (We verified that the strain was a valid knockout using primers, but it is possible that the phenotype results from extraneous mutations.) |
| *MET31 + MET32* | *ORF19.1757* | | *scMET31+scMET32*: Methionine auxotrophy[32]* | Methionine auxotrophy is only seen in the double knockout. |
| *RLM1 + SMP1* | *RLM1* | | *scRLM1*: Caffeine and Rapamycin sensitivity*§ and Calcofluor White resistance[33]; *caRLM1*: Calcofluor White sensitivity* | No *scSMP1* deletion mutant was available to assay phenotypes. |
| *Mixed Similarity Orthologs* | | | | |
| *CAD1 + YAP1* | *CAP1* | *scCAD1 + caYAP1:* Oxidative stress sensitivity[34,35]* | *scYAP1*: Fenpropimorph sensitivity*§ | |
| *Similar Orthologs* | | | | |
| *ACE2 + SWI5*¥ | *ACE2* | *scACE2 + caACE2*: Cell separation defect[36,37]*§ | | The *scSWI5* regulator regulates homothallic switching[36]. |
| *ACA1 + CST6* | *ORF19.6102* | *scCST6 + caORF19.6102*: Impaired growth on base media[3]*§ | | |
| *CUP2 + HAA1* | *CUP2* | *scCUP2 + caCUP2*: Copper sensitivity*§ | | |
| *SKN7 + HMS2* | *SKN7* | *scSKN7 + caSKN7*: $H_2O_2$ sensitivity[31,38]* | | |

¥ *scACE2* was identified as the ortholog of *caACE2* in CGD, but the WGD gene *scSWI5* was missed (both were missed by SYNERGY)

† These genes are classified as orthologs using SYNERGY but are classified as "Best Hits" in CGD.

‡ These genes are classified as orthologs in CGD but lacked a clear ortholog using SYNERGY

**C. albicans* phenotype assayed in this study

§ *S. cerevisiae* phenotype assayed in this study

## References

1. Johnston M (1987) A model fungal gene regulatory mechanism: the GAL genes of Saccharomyces cerevisiae. Microbiol Rev 51: 458-476.
2. Martchenko M, Levitin A, Hogues H, Nantel A, Whiteway M (2007) Transcriptional rewiring of fungal galactose-metabolism circuitry. Curr Biol 17: 1007-1013.
3. Deutschbauer AM, Jaramillo DF, Proctor M, Kumm J, Hillenmeyer ME, et al. (2005) Mechanisms of haploinsufficiency revealed by genome-wide profiling in yeast. Genetics 169: 1915-1925.
4. Akache B, Wu K, Turcotte B (2001) Phenotypic analysis of genes encoding yeast zinc cluster proteins. Nucleic Acids Res 29: 2181-2190.
5. Giaever G, Chu AM, Ni L, Connelly C, Riles L, et al. (2002) Functional profiling of the Saccharomyces cerevisiae genome. Nature 418: 387-391.
6. Small WC, Brodeur RD, Sandor A, Fedorova N, Li G, et al. (1995) Enzymatic and metabolic studies on retrograde regulation mutants of yeast. Biochemistry 34: 5569-5576.
7. Dubois E, Messenguy F (1985) Isolation and characterization of the yeast ARGRII gene involved in regulating both anabolism and catabolism of arginine. Mol Gen Genet 198: 283-289.
8. Arndt KT, Styles C, Fink GR (1987) Multiple global regulators control HIS4 transcription in yeast. Science 237: 874-880.
9. Liao WL, Ramon AM, Fonzi WA (2008) GLN3 encodes a global regulator of nitrogen metabolism and virulence of C. albicans. Fungal Genet Biol 45: 514-526.
10. Dudley AM, Janse DM, Tanay A, Shamir R, Church GM (2005) A global view of pleiotropy and phenotypically derived gene function in yeast. Mol Syst Biol 1: 2005 0001.
11. Shankar CS, Ramakrishnan MS, Umesh-Kumar S (1996) MIG1 overexpression causes flocculation in Saccharomyces cerevisiae. Microbiology 142 ( Pt 9): 2663-2667.
12. Braun BR, Johnson AD (2000) TUP1, CPH1 and EFG1 make independent contributions to filamentation in candida albicans. Genetics 155: 57-67.
13. Kim MJ, Kil M, Jung JH, Kim J (2008) Roles of Zinc-responsive transcription factor Csr1 in filamentous growth of the pathogenic Yeast Candida albicans. J Microbiol Biotechnol 18: 242-247.
14. Godard P, Urrestarazu A, Vissers S, Kontos K, Bontempi G, et al. (2007) Effect of 21 different nitrogen sources on global gene expression in the yeast Saccharomyces cerevisiae. Mol Cell Biol 27: 3065-3086.
15. Xie MW, Jin F, Hwang H, Hwang S, Anand V, et al. (2005) Insights into TOR function and rapamycin response: chemical genomic profiling by using a high-density cell array method. Proc Natl Acad Sci U S A 102: 7215-7220.
16. Marvin ME, Mason RP, Cashmore AM (2004) The CaCTR1 gene is required for high-affinity iron uptake and is transcriptionally controlled by a copper-sensing transactivator encoded by CaMAC1. Microbiology 150: 2197-2208.
17. Jungmann J, Reins HA, Lee J, Romeo A, Hassett R, et al. (1993) MAC1, a nuclear regulatory protein related to Cu-dependent transcription factors is involved in Cu/Fe utilization and stress resistance in yeast. Embo J 12: 5051-5056.

18. Som I, Mitsch RN, Urbanowski JL, Rolfes RJ (2005) DNA-bound Bas1 recruits Pho2 to activate ADE genes in Saccharomyces cerevisiae. Eukaryot Cell 4: 1725-1735.
19. Moreno I, Pedreno Y, Maicas S, Sentandreu R, Herrero E, et al. (2003) Characterization of a Candida albicans gene encoding a putative transcriptional factor required for cell wall integrity. FEMS Microbiol Lett 226: 159-167.
20. Sexton JA, Brown V, Johnston M (2007) Regulation of sugar transport and metabolism by the Candida albicans Rgt1 transcriptional repressor. Yeast 24: 847-860.
21. Marshall-Carlson L, Neigeborn L, Coons D, Bisson L, Carlson M (1991) Dominant and recessive suppressors that restore glucose transport in a yeast snf3 mutant. Genetics 128: 505-512.
22. Lamb TM, Xu W, Diamond A, Mitchell AP (2001) Alkaline response genes of Saccharomyces cerevisiae and their relationship to the RIM101 pathway. J Biol Chem 276: 1850-1856.
23. Davis D, Wilson RB, Mitchell AP (2000) RIM101-dependent and-independent pathways govern pH responses in Candida albicans. Mol Cell Biol 20: 971-978.
24. Silver PM, Oliver BG, White TC (2004) Role of Candida albicans transcription factor Upc2p in drug resistance and sterol metabolism. Eukaryot Cell 3: 1391-1397.
25. MacPherson S, Akache B, Weber S, De Deken X, Raymond M, et al. (2005) Candida albicans zinc cluster protein Upc2p confers resistance to antifungal drugs and is an activator of ergosterol biosynthetic genes. Antimicrob Agents Chemother 49: 1745-1752.
26. Crowley JH, Leak FW, Jr., Shianna KV, Tove S, Parks LW (1998) A mutation in a purported regulatory gene affects control of sterol uptake in Saccharomyces cerevisiae. J Bacteriol 180: 4177-4183.
27. Andre B (1990) The UGA3 gene regulating the GABA catabolic pathway in Saccharomyces cerevisiae codes for a putative zinc-finger protein acting on RNA amount. Mol Gen Genet 220: 269-276.
28. Bauer J, Wendland J (2007) Candida albicans Sfl1 suppresses flocculation and filamentation. Eukaryot Cell 6: 1736-1744.
29. Li Y, Su C, Mao X, Cao F, Chen J (2007) Roles of Candida albicans Sfl1 in hyphal development. Eukaryot Cell 6: 2112-2121.
30. Robertson LS, Fink GR (1998) The three yeast A kinases have specific signaling functions in pseudohyphal growth. Proc Natl Acad Sci U S A 95: 13783-13787.
31. Hillenmeyer ME, Fung E, Wildenhain J, Pierce SE, Hoon S, et al. (2008) The chemical genomic portrait of yeast: uncovering a phenotype for all genes. Science 320: 362-365.
32. Blaiseau PL, Isnard AD, Surdin-Kerjan Y, Thomas D (1997) Met31p and Met32p, two related zinc finger proteins, are involved in transcriptional regulation of yeast sulfur amino acid metabolism. Mol Cell Biol 17: 3640-3648.
33. Dodou E, Treisman R (1997) The Saccharomyces cerevisiae MADS-box transcription factor Rlm1 is a target for the Mpk1 mitogen-activated protein kinase pathway. Mol Cell Biol 17: 1848-1859.
34. Stephen DW, Rivers SL, Jamieson DJ (1995) The role of the YAP1 and YAP2 genes in the regulation of the adaptive oxidative stress responses of Saccharomyces cerevisiae. Mol Microbiol 16: 415-423.
35. Alarco AM, Raymond M (1999) The bZip transcription factor Cap1p is involved in multidrug resistance and oxidative stress response in Candida albicans. J Bacteriol 181: 700-708.

36. Dohrmann PR, Butler G, Tamai K, Dorland S, Greene JR, et al. (1992) Parallel pathways of gene regulation: homologous regulators SWI5 and ACE2 differentially control transcription of HO and chitinase. Genes Dev 6: 93-104.
37. Mulhern SM, Logue ME, Butler G (2006) Candida albicans transcription factor Ace2 regulates metabolism and is required for filamentation in hypoxic conditions. Eukaryot Cell 5: 2001-2013.
38. Singh P, Chauhan N, Ghosh A, Dixon F, Calderone R (2004) SKN7 of Candida albicans: mutant construction and phenotype analysis. Infect Immun 72: 2390-2394.
